# Supplementary material for: Neurocognitive characterization of behaviour and mental illness through time-varying brain network analysis
Source: Nat Commun. 2026 Feb 3;17:1353. doi: 10.1038/s41467-025-67398-w (PMC12877127; doi:10.1038/s41467-025-67398-w)
Supplement: Supplementary file 1 — Supplementary Information [file 41467_2025_67398_MOESM1_ESM.pdf]

Supplementary Materials for

Neurocognitive characterization of behaviour and mental illness through time-varying brain network analysis

This file includes:

Supplementary Text

Figs. S1 to S24

Tables S1 to S14

Supplementary Text

Design of fMRI tasks

Emotional faces task (EFT): The EFT was designed by Grosbras and Paus (2006) to assess social-emotional processing [1](Fig. S1). Participants were instructed to watch a 18s block of either face (angry, happy, neutral) or non-face (control) stimuli. Each block of face stimuli comprises black and white video clips (2-5 s) of one type of face in movement (three males and three females). The control stimuli block consists of black and white concentric circles expanding or contracting at various speeds, roughly matching the contrast and motion of the face clips. Each type of face stimuli was repeated four times and intermixed with twelve blocks of the control stimuli. Task stimuli onset and duration were the same across all subjects. Total scanning session of the EFT lasted about 7 minutes (197 fMRI scans).

Monetary incentive delay task (MID): The MID was modified based on Knutson et al., (2001) to investigate neural mechanism of reward processing [2]. Scanning session consists of 42 10s trials for every participant. Each trial begins with an anticipatory cue (250 ms), indicating participant can win 10 points (large-win), or 2 points (small-win) or no points (no-win) if responded correctly. After a variable delay (4,000-4,500 ms) of fixation on a white cross-hair, a target (250-400 ms) appears on the left or right side of the screen the same as the cue. Participants were instructed to press the left or right button using the index finger corresponding to the side of target as soon as possible. The duration of target was automatically adjusted to make each participant has about 66% successful response rate [2]. Feedback was presented 1,450 ms after the response to show how many points participants received in this trial. Each trial lasts 10 seconds, and the sequence of trials were the same across subjects (42 trials in total). Scanning session of the MID takes about 7 minutes (192 scans).

Stop-signal task (SST): The SST was designed to measure neural response of inhibition control [3]. Participants were instructed to press button using the left or right index finger corresponding to go signals (an arrow pointing to left or right, 60 trials), and withhold their responses to unpredictable stop signals (an arrow pointing upwards immediately followed the go signals, 300 trials). Duration of go signals is 1000 ms and duration of for stop signal varies between 0 – 900 ms, which is automatically adjusted using a tracking algorithm to let participants have 50% successful and 50% unsuccessful inhibition trials [3]. Total scanning session contains 310 ~ 350 scans for every participant.

Fig. S1 Design of fMRI tasks were based on previous studies: a) emotional faces task (EFT) [1]; b) monetary incentive delay task (MID) [2]; c) stop-signal task (SST) [3]. In the EFT, real human facial images depicting happy, neutral, and angry expressions were presented as stimuli.

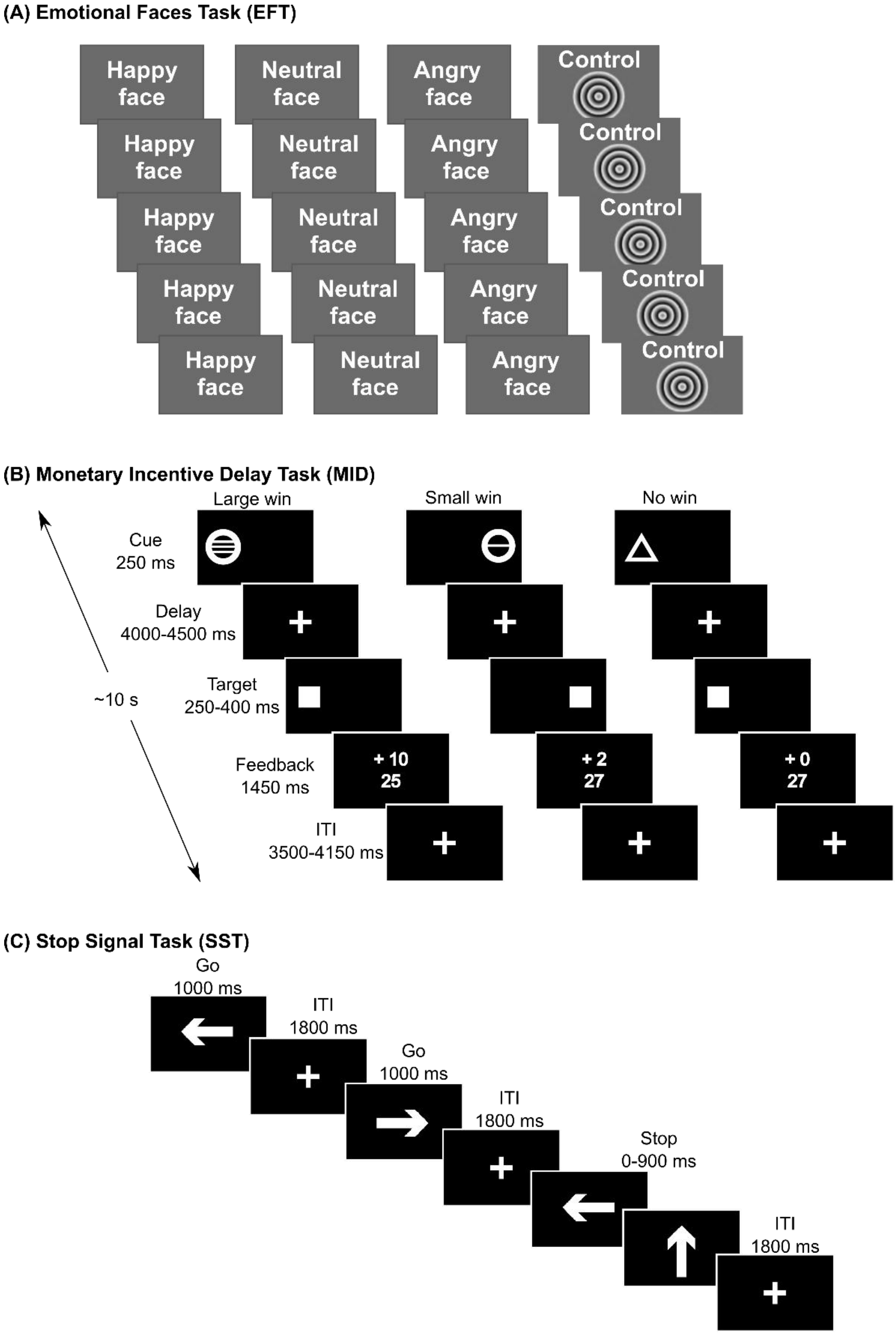

Fig. S2. Sixty-one components corresponding to grey matter areas were identified with group independent component analysis (ICA) for resting-state and task-fMRI sessions. These components were categorized into seven domains based on their functions and anatomical locations: subcortical (SCN), temporal (TEP), sensorimotor (SMN), visual (VSN), cognitive control (CON), default mode (DMN) and cerebellar (CEB) networks.

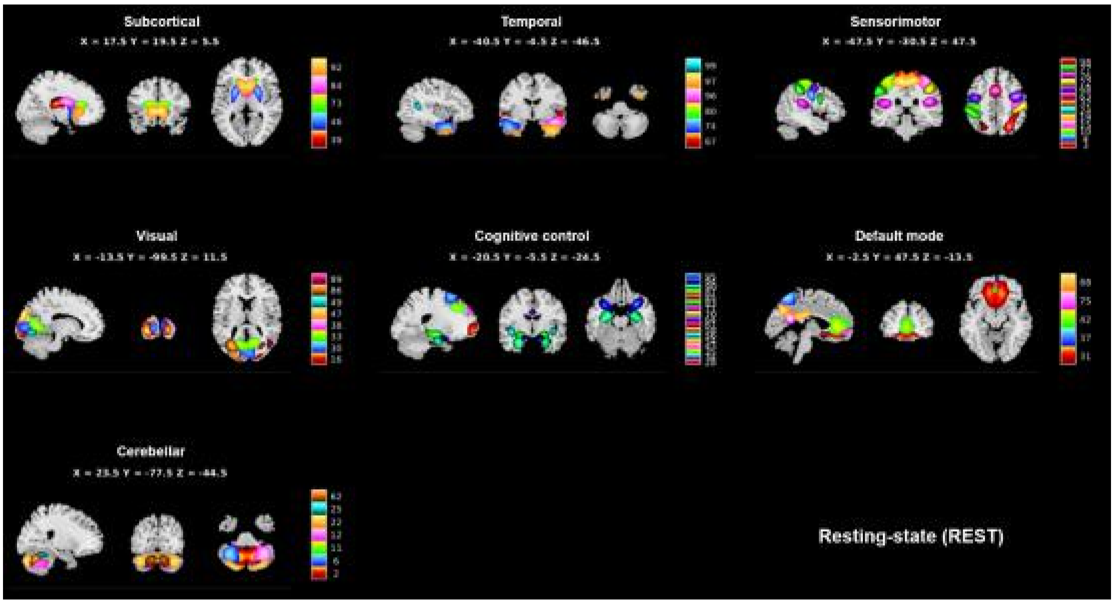

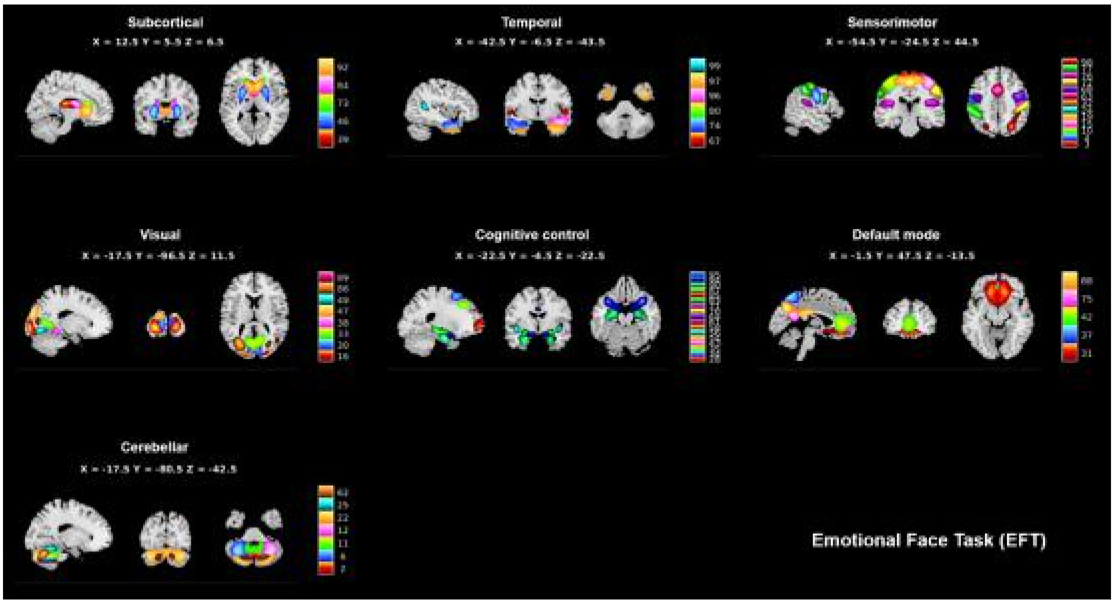

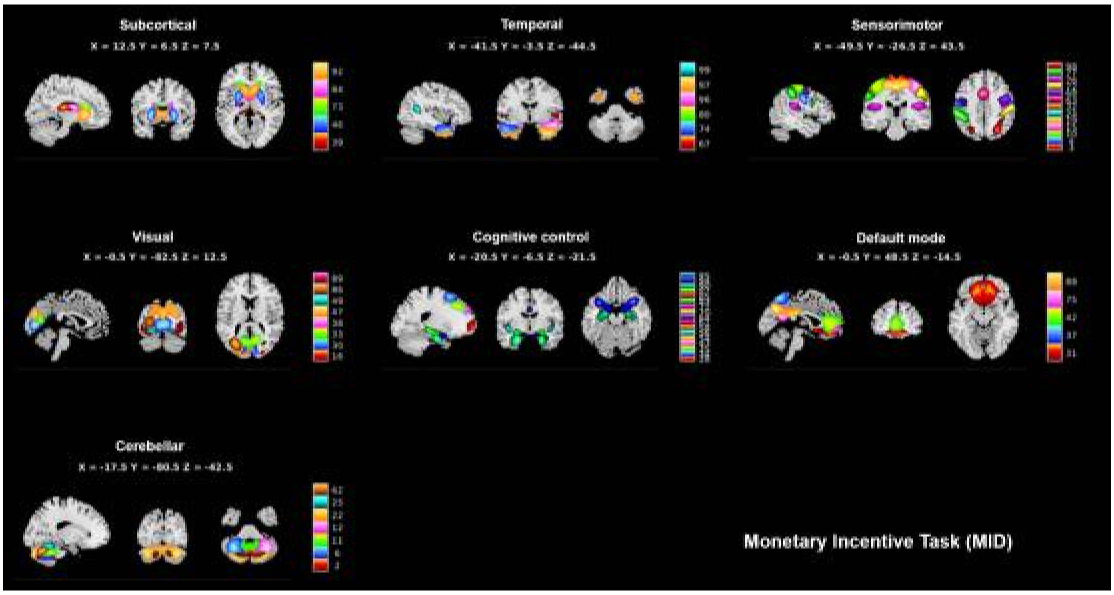
Subcortical
X = 12.5 Y = 6.5 Z = 7.5
Temporal
X = -41.5 Y = -3.5 Z = -44.5
Sensorimotor
X = -49.5 Y = -26.5 Z = 43.5
Visual
X = -0.5 Y = -82.5 Z = 12.5
Cognitive control
X = -20.5 Y = -6.5 Z = -21.5
Default mode
X = -0.5 Y = 48.5 Z = -14.5
Cerebellar
X = -17.5 Y = -80.5 Z = -42.5
Monetary Incentive Task (MID)

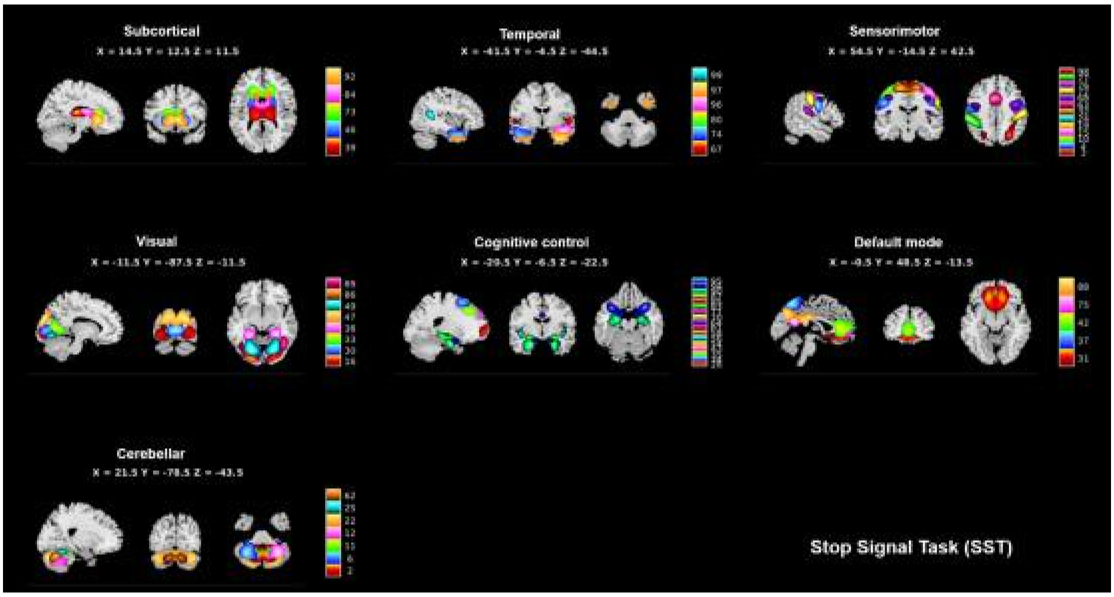
Subcortical
X = 14.5 Y = 12.5 Z = 11.5
Temporal
X = -41.5 Y = -4.5 Z = -44.5
Sensorimotor
X = 56.5 Y = -14.5 Z = 42.5
Visual
X = -11.5 Y = -87.5 Z = -11.5
Cognitive control
X = -20.5 Y = -6.5 Z = -22.5
Default mode
X = -0.5 Y = 48.5 Z = -13.5
Cerebellar
X = 21.5 Y = -78.5 Z = -43.5
Stop Signal Task (SST)

Fig. S3 Optimal number of *k*-means clusters estimated using the elbow criterion for the a) resting-state; b) emotional face task; c) monetary incentive task; and d) stop signal task fMRI sessions. The x-axis is the cluster number varying from 2 to 10 in different scanning sessions. The y-axis represents the within-cluster sum of squares divided by between-cluster sum of squares.

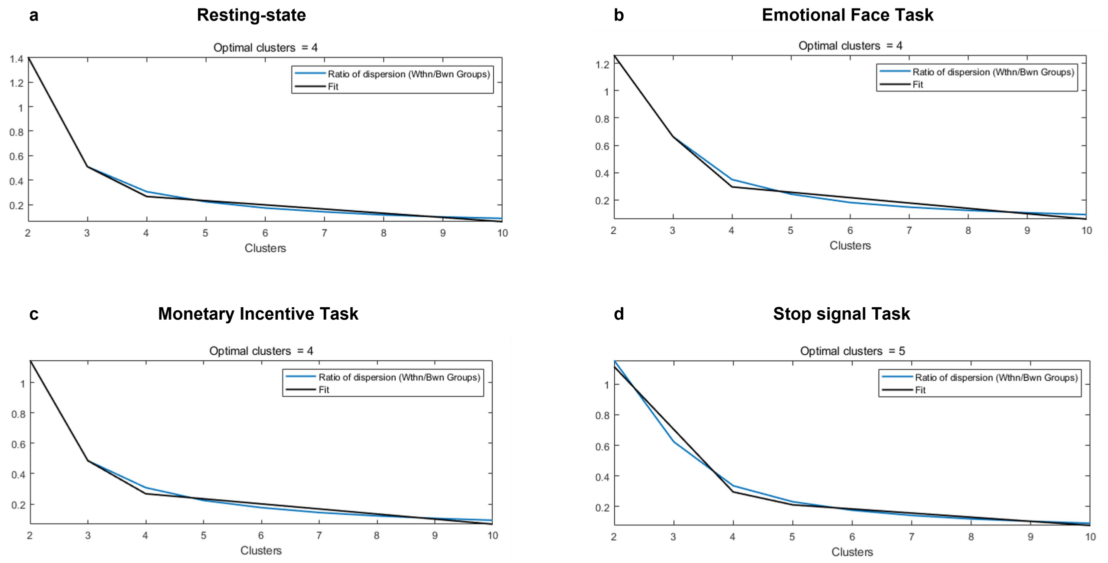

Fig. S4 Connectogram plot of the FNC states for the resting-state (REST) and three task-fMRI sessions (EFT, MID, SST). The strongest 100 connectivity of each state were shown.

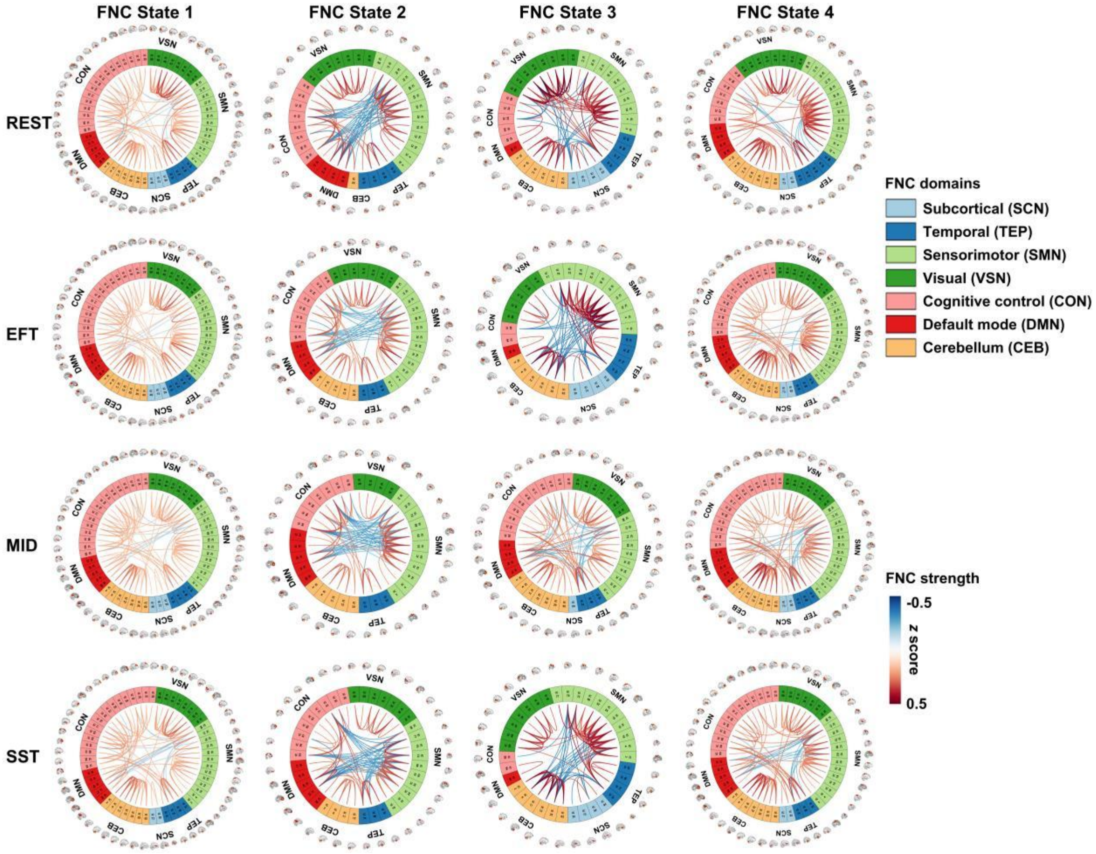

Fig. S5 FNC states were reproducible with different sliding window lengths from 8.8s (4TR) to 70.4s (32TR). FNC with the highest correlation with the FNC states using window length = 17.6s (8TR) were plotted in the same color frame.

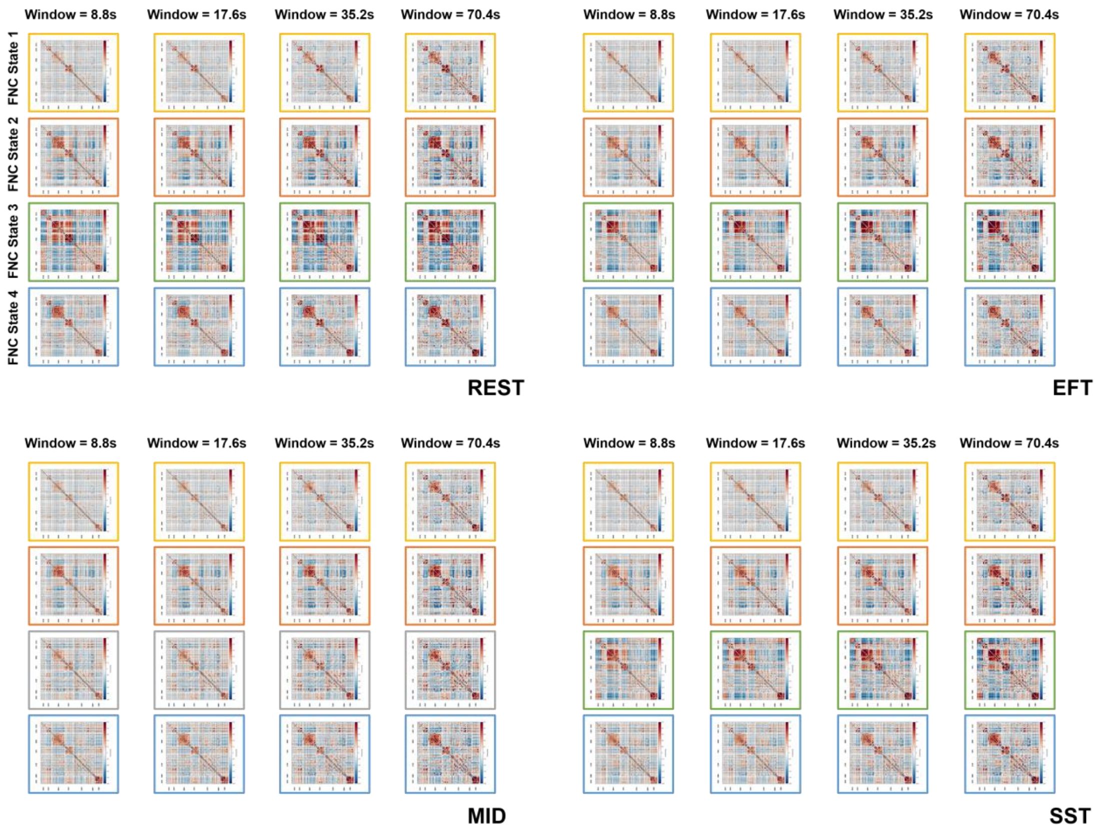

Fig. S6 Correlation between dwell time of FNC states with different sliding window lengths. For window lengths of 8.8s (4TR), 35.2 (16TR) and 70.4s (32TR), FNC states' dwell time are correlated with the corresponding FNC states' dwell time using window length of 17.6s (8TR). Pearson's correlation coefficient and significance are shown for each plot. Different colors indicate different FNC states.

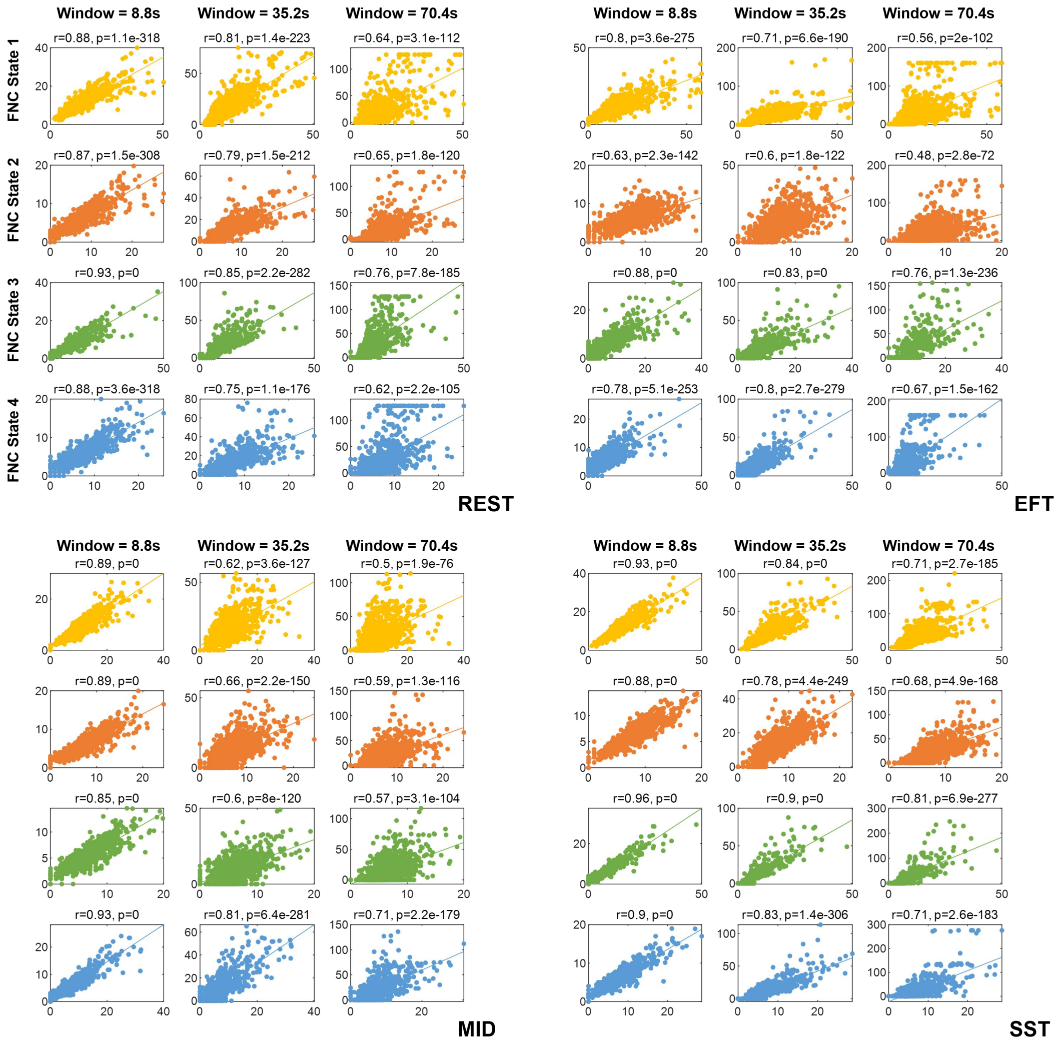

Fig. S7 FNC states with different *k*-means clustering number. For $k = 2, 3, 5$, FNC states with the highest correlation with FNC states using $k = 4$ were plotted in the same color frame.

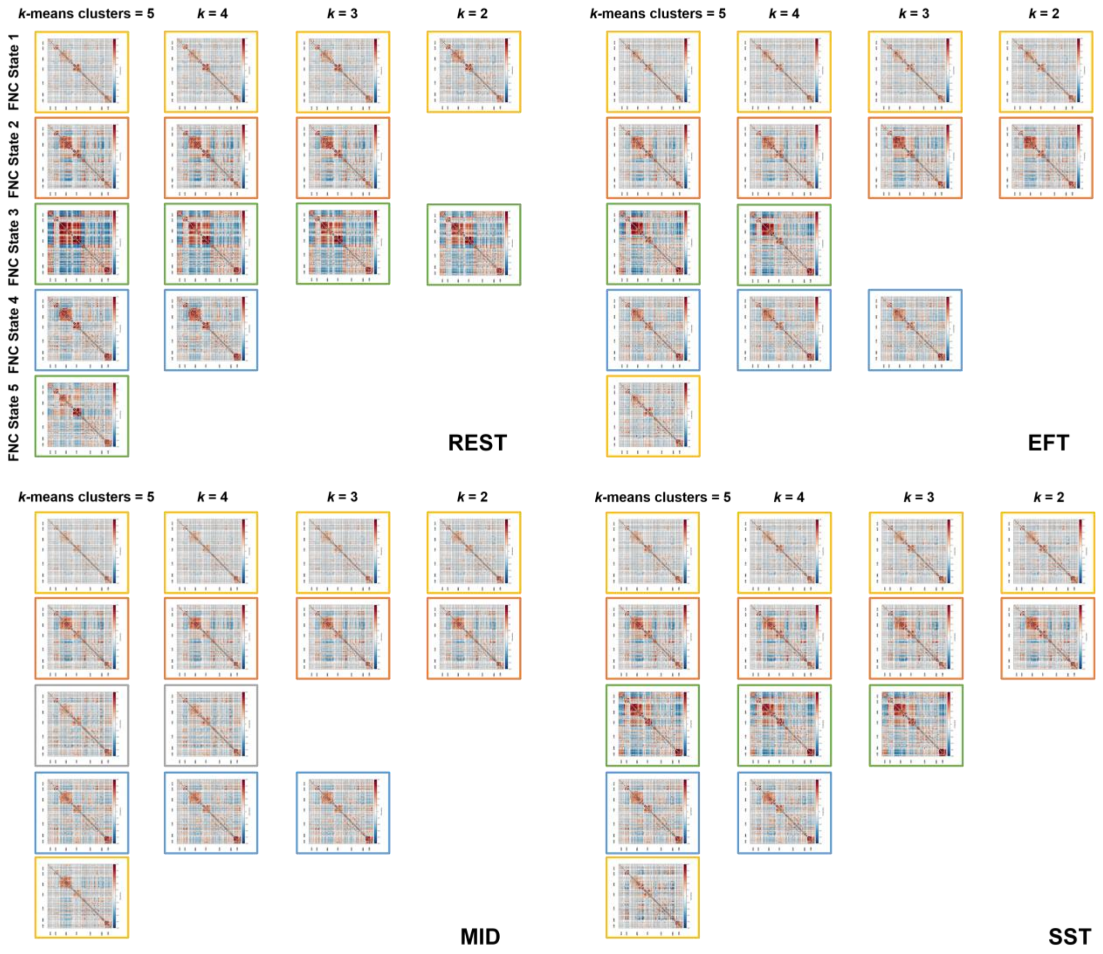

Fig. S8 Correlation between dwell time of FNC states with different *k*-means clustering number. For $k = 2, 3, 5$, FNC states' dwell time correlated with the corresponding FNC states' dwell time using $k = 4$. Pearson's correlation coefficient and significance are shown for each plot. Different colors indicate different FNC states.

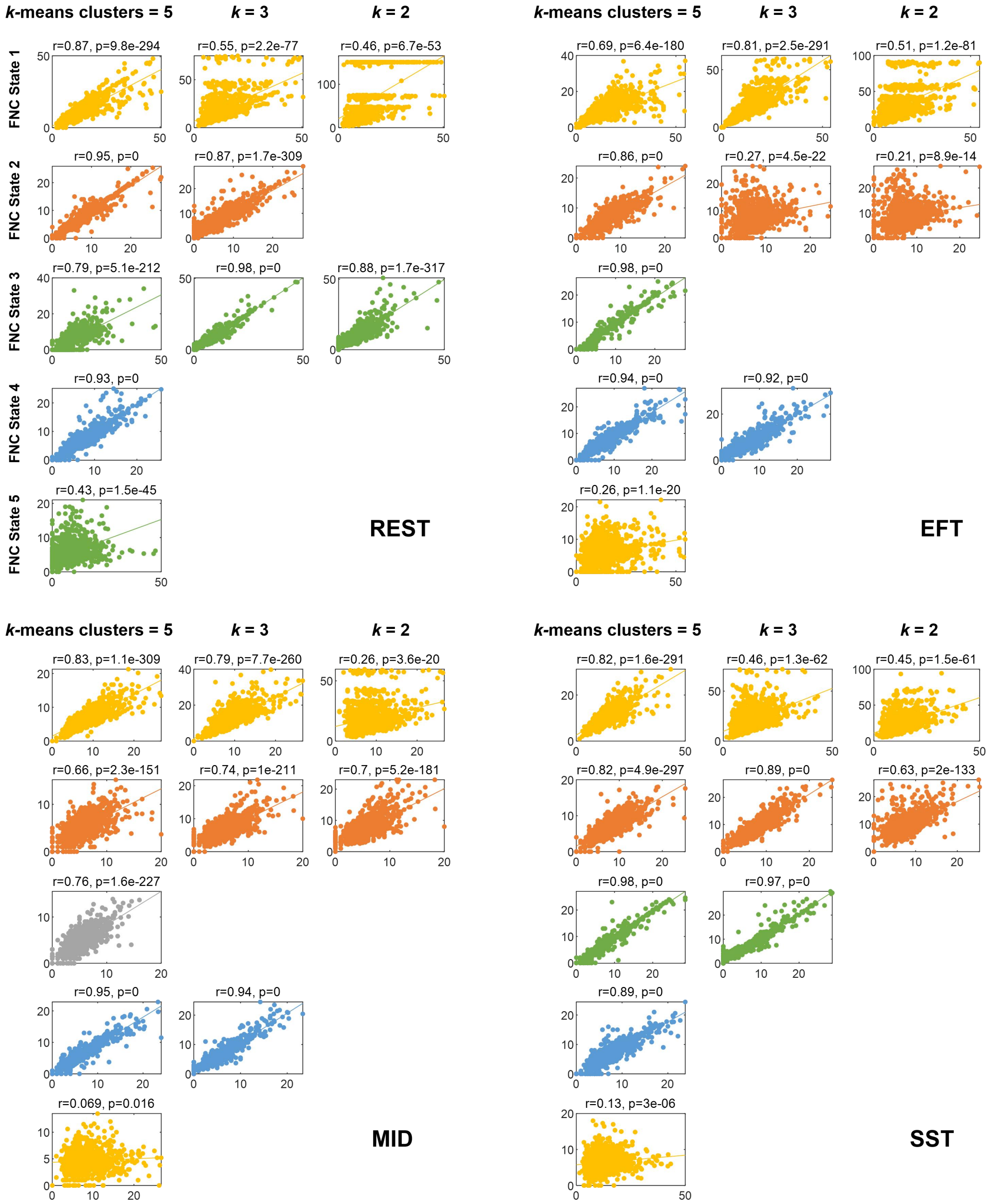

Fig. S9 Distribution of MID and SST task performances of all participants.

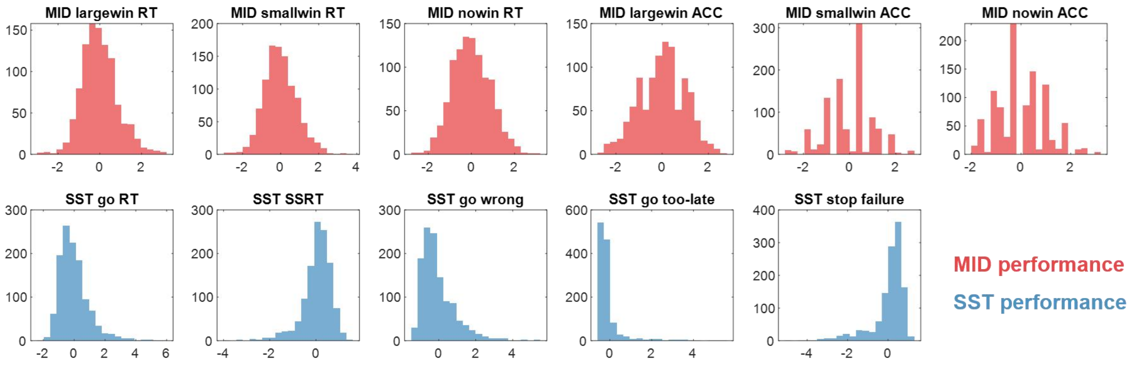

Fig. S10 Spearman's correlation between FNC state dwell time and task performances in MID and SST. After FDR, SST go too-late error showed a positive association with state 3 (Spearman's *rho*=0.42, p=9.03x10^{-24}) and a negative association with state 1 (*rho*=-0.10, p=9.29x10^{-4}).

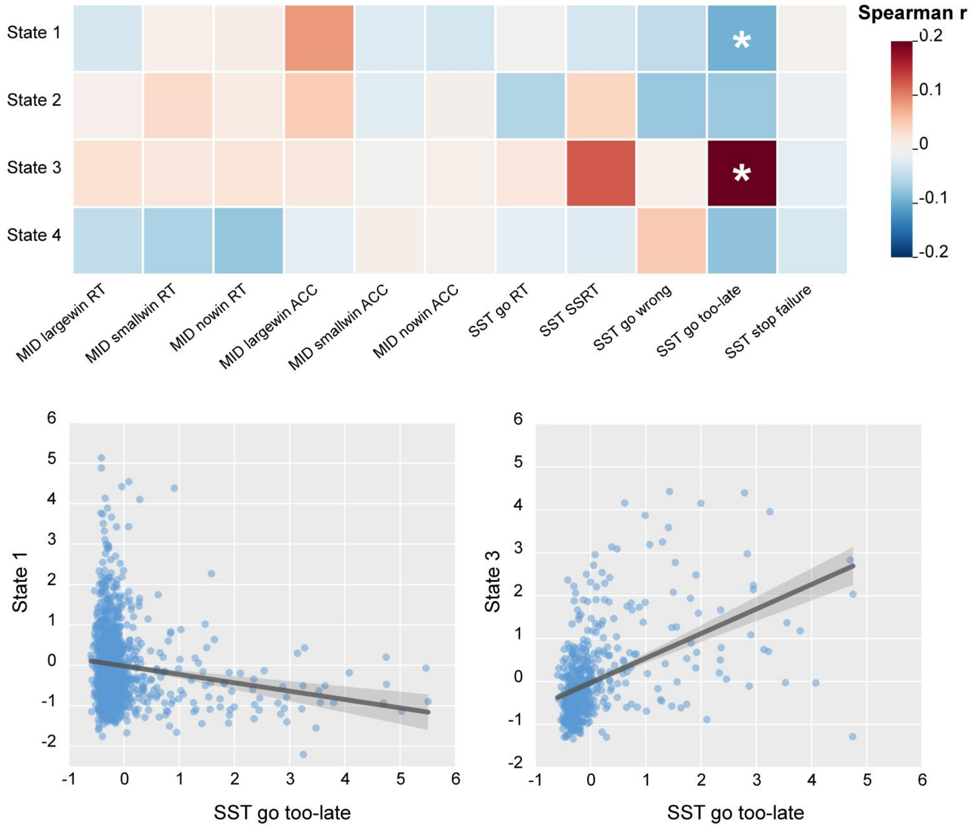

Fig. S11 FNC state occurrence using window length of 8.8s and 17.6s. The almost identical state occurrence fluctuation between different window lengths suggests that FNC state occurrence was not driven by the choice of window length.

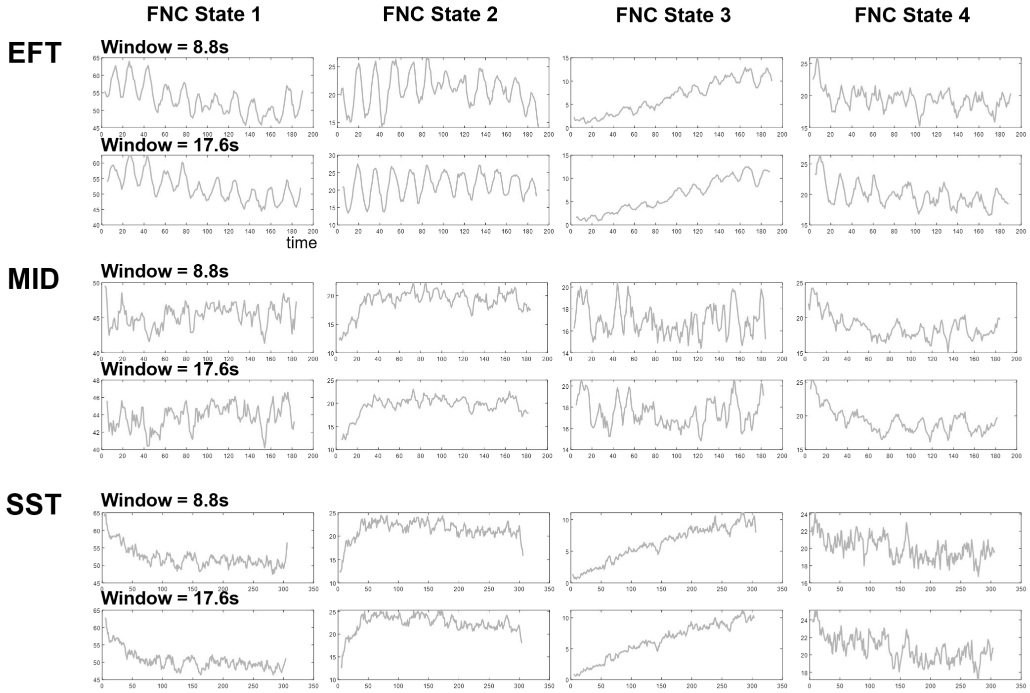

Fig. S12 Distribution of reinforcement-related behaviour questionnaires of all participants. Full names of the items were listed in Table S9.

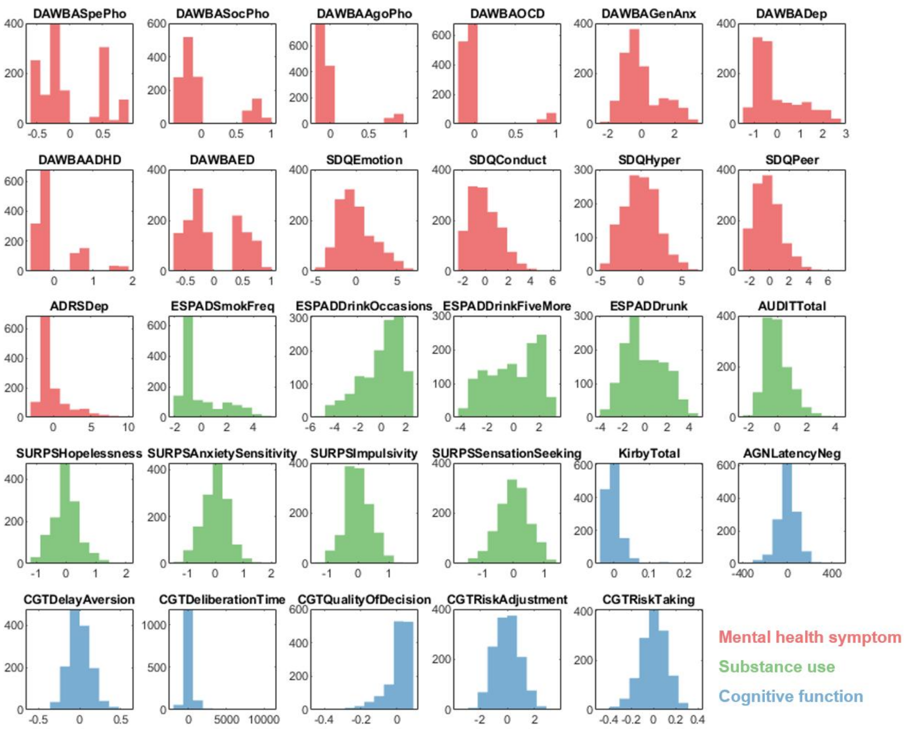

Fig. S13 Adjusted R^2 of 23 reinforcement-related behaviours regressed by time-varying or static FNC or both in IMAGEN.

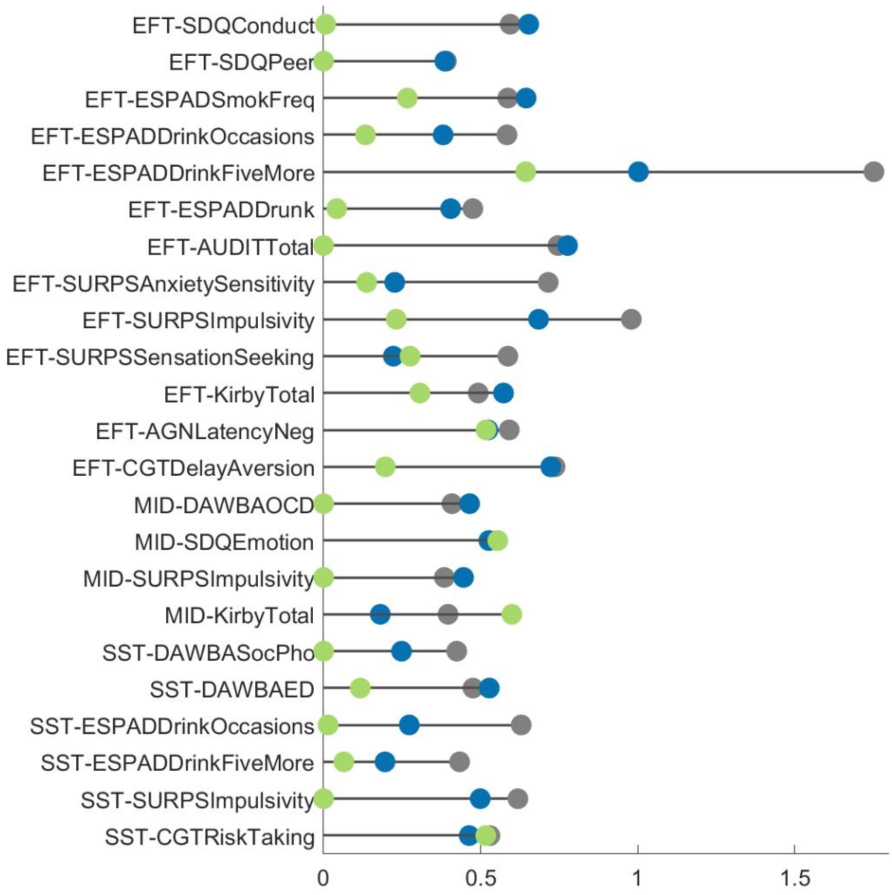

Fig. S14 FNC states derived from the STRATIFY cohort and their spatial correlations with FNC states derived from the IMAGEN resting-state (upper row). Pearson's correlation between FNC matrices of the same state ranges from 0.80 ~ 0.96, showing a high-level of correspondence. Similar to the IMAGEN dataset, we couldn't extract the FNC state 3 from the MID session as other sessions.

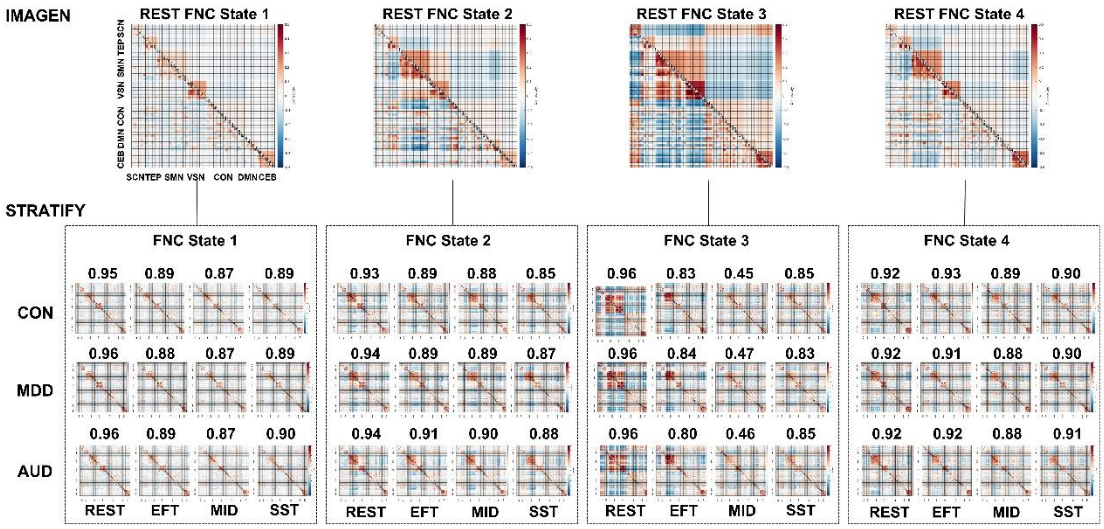

Fig. S15 Correlation between FNC state occurrence and task conditions in STRATIFY CON, MDD and AUD groups. For the a) EFT and b) MID, Pearson correlation between state occurrences and task stimuli were calculated at the group level similar to the main analysis (Figure 3). c) For the SST, we calculated the partial correlation between state occurrence and task stimuli (controlling for other types of stimuli) on the individual level. Asterisks indicate FDR-corrected statistical significance across the three tasks.

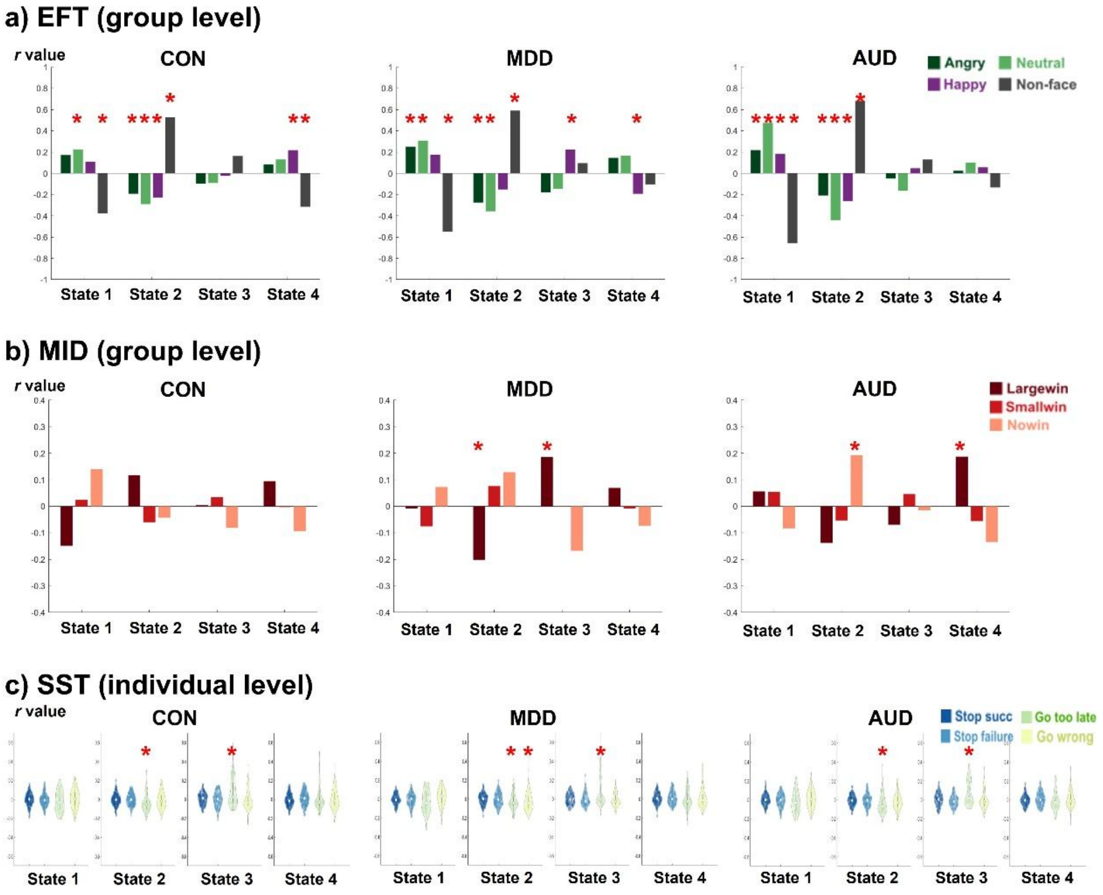

Fig. S16 MID and SST task performances of STRTIFY CON, MDD and AUD patients. Red lines indicate significant group differences after FDR correction.

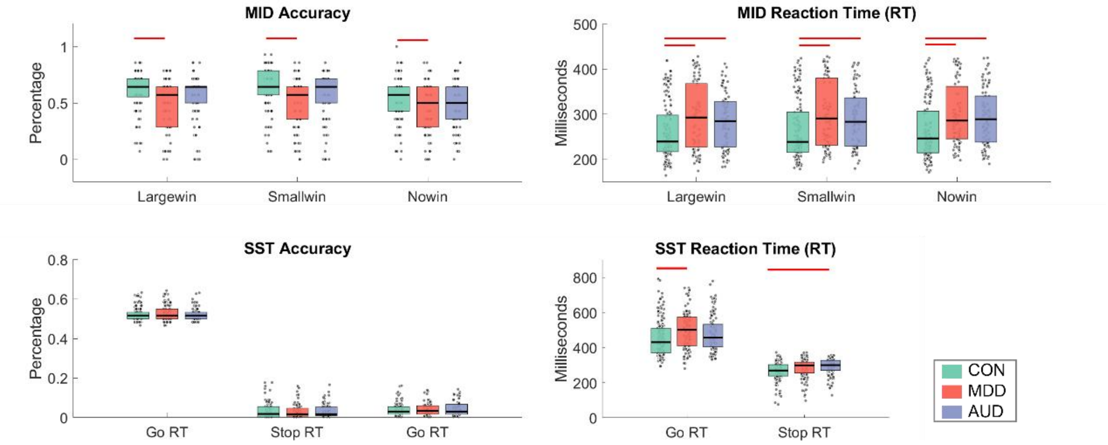

Fig. S17 An example of R^2 and adjusted R^2 changes with sample size by randomly sampling subjects from the IMAGEN cohort. We regressed the SDQ peer problem score on the full model (time-varying FNC and static FNC) in the same way as described in section ***Time-varying FNC explains more behavioural variance than static FNC***. With each sample size, we repeated the regression analysis 50 times. As sample size increases, both R^2 and adjusted R^2 converges and showing smaller variances.

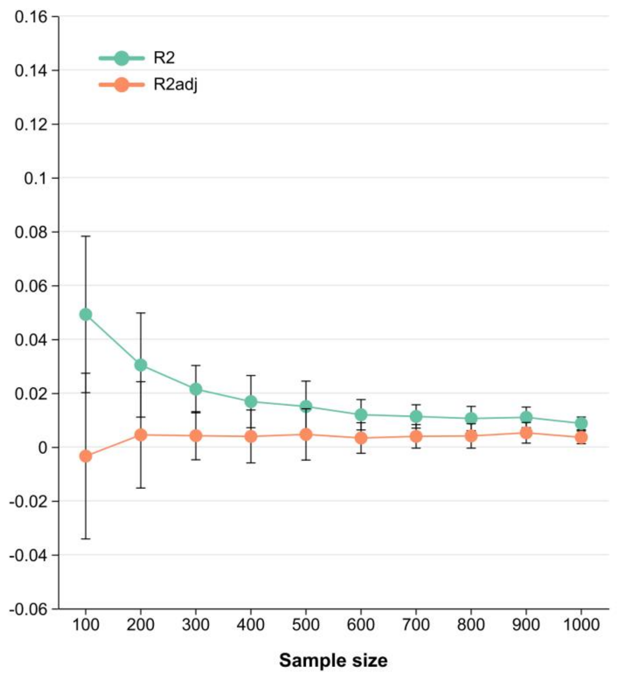

Fig. S18 Post-hoc group comparison of connectivity strength of time-varying FNC and static FNC between CON, MDD, and AUD patients. Two-sample *t*-tests were performed between groups to compare the connectivity strength of each state in time-varying FNC and static FNC across groups. Red lines indicate significant differences after FDR correction for multiple comparisons.

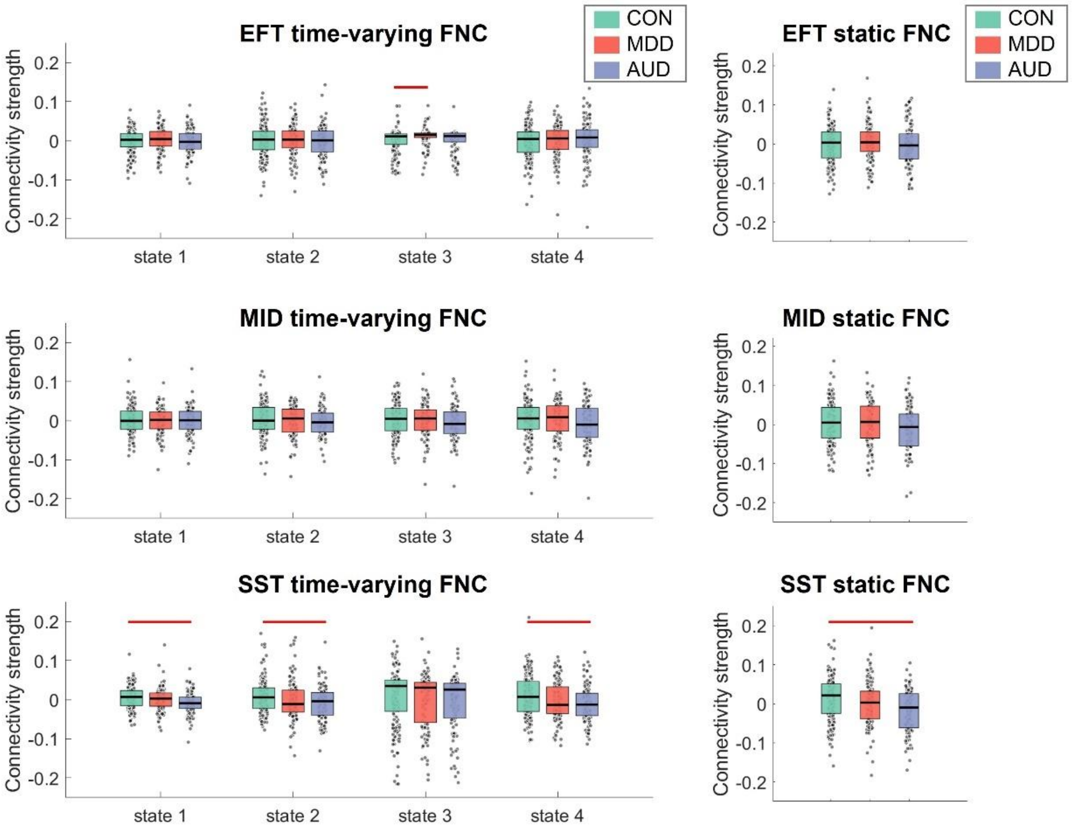

Fig. S19 Influence of recruitment site effect on FNC states. a) Four FNC states were identified separately for each of the eight recruitment sites. As an example, the cluster centroids of FNC states from recruitment site 1 are shown. b) To quantitatively assess the similarity of FNC states across sites, we computed the spatial correlation between the FNC states derived from each site and those derived from all sites combined. The bar plot shows the mean correlation for each site, represented in different colors. Mean correlation values are indicated above each bar, and error bars represent the standard deviation across the four states. The results demonstrate that FNC states derived from individual sites were highly similar to those from all sites combined. c) Correlation of FNC state occurrences between each individual site and those from all sites combined. For each site, the mean and standard deviation of the correlations across the four states are shown. Although there was greater variability in state occurrence correlations across sites, the mean correlations ranged from 0.34 to 0.82, indicating moderate to large consistency.

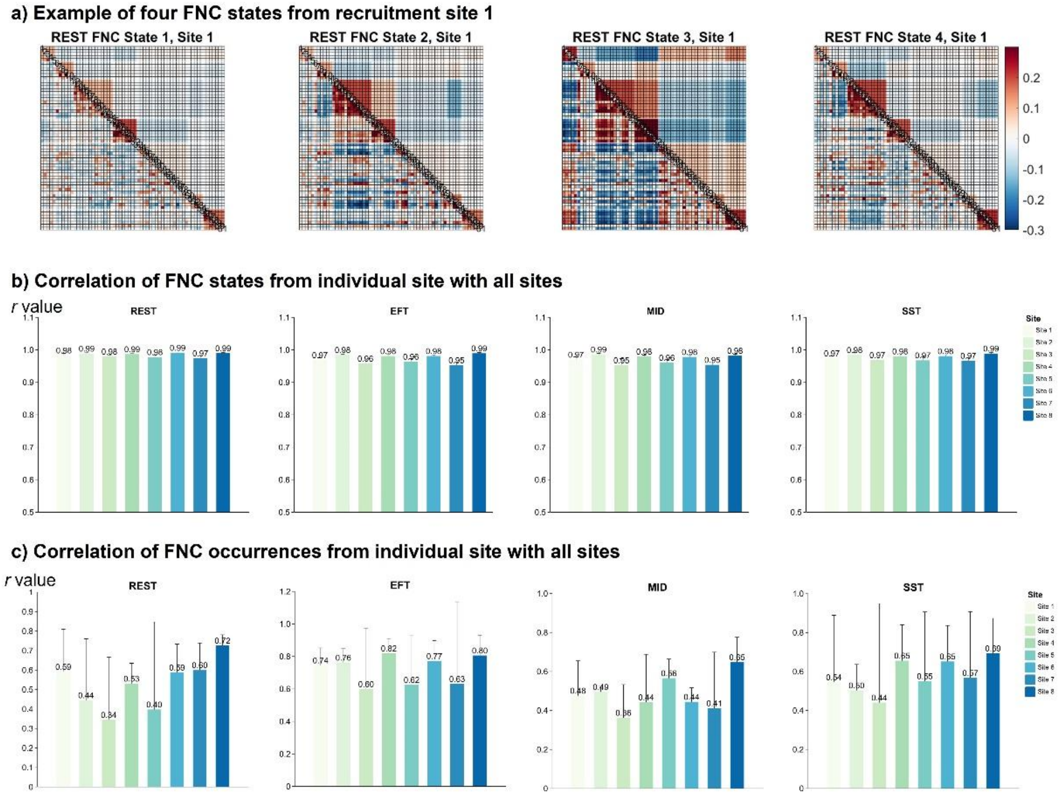

Fig. S20 Replication of the correlation between FNC state occurrence and task conditions across recruitment sites for the a) emotional face task (EFT); b) monetary incentive task (MID) and c) stop signal task (SST). Correlation coefficients (r values) were computed using the same method as in the main results shown in Figure 3. The bar plot displays the mean and standard deviation of the correlation coefficients for each site, with individual site-level values represented as dots. A fixed-effect meta-analysis was conducted to assess the overall significance across sites. Asterisks indicate FDR-corrected statistical significance across all three tasks and all conditions.

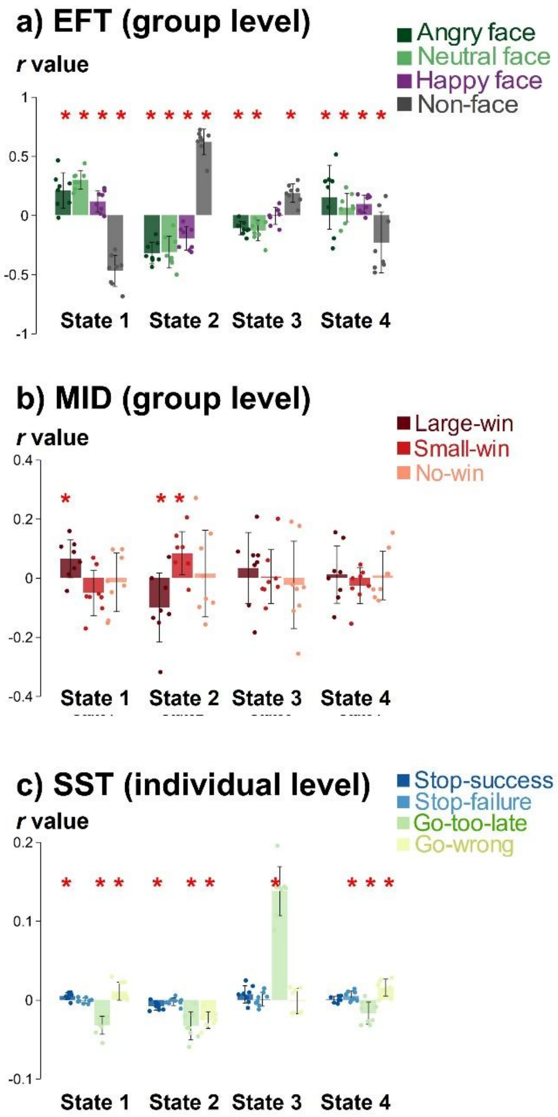

Fig. S21 Replication of the correlation between FNC state dwell time and task performance across recruitment sites. The main results (Fig. S10) showed that go-too-late errors in the SST task were positively associated with dwell time in state 3 and negatively associated with dwell time in state 1. Correlation coefficients were computed separately for each recruitment site. The bar plot displays the mean and standard deviation of the correlation coefficients across sites, with individual site-level values represented as dots. A fixed-effect meta-analysis was conducted to assess the overall significance across sites. Asterisks indicate FDR-corrected statistical significance.

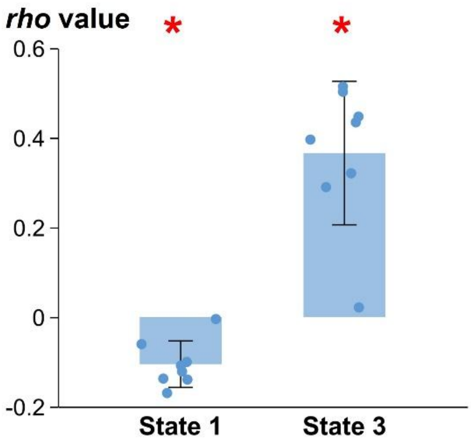

Fig. S22 Replication of behavioural variance explained by time-varying and static FNC across recruitment sites. Using linear regression models, we assessed the variance explained (R^2) by time-varying FNC (blue) and static FNC (green) using linear regression model across the 23 behavioral items, as reported in the main results (Figure 6). The bar plot shows the mean and standard deviation of the R^2 values across the recruitment sites.

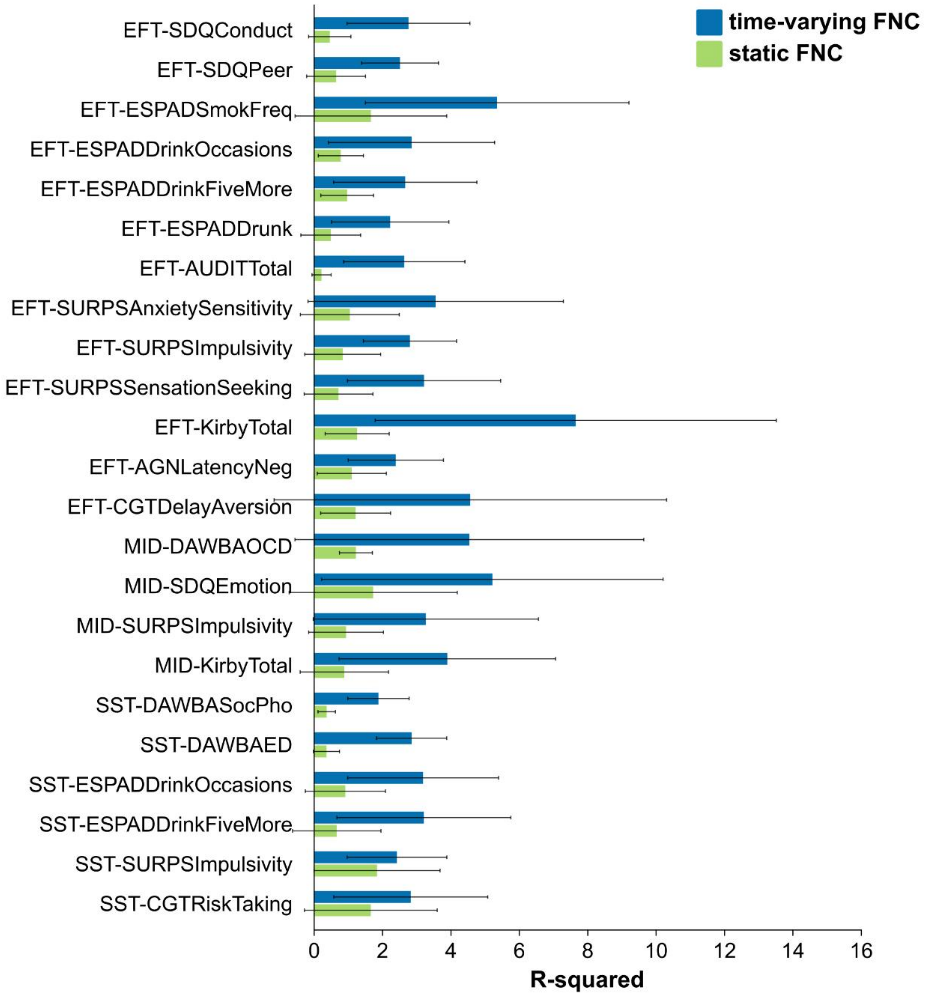

Fig. S23 Correlation between subjects' head motion (framewise displacement, FD) and state occurrences during each scanning session.

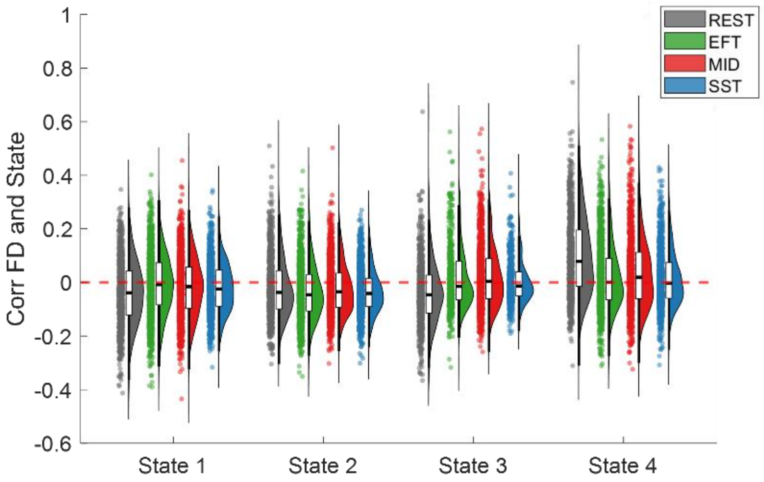

Fig. S24 Replication of the correlation between FNC state occurrence and task conditions with head motion (FD) added as an additional regressor using partial correlation. Results are shown for the: a) emotional face task (EFT); b) monetary incentive task (MID) and c) stop signal task (SST). Correlation coefficients (r values) were computed using the same method as in the main results shown in Figure 3. Asterisks indicate FDR-corrected statistical significance across the three tasks. For EFT and SST, we replicated all significant results in the main analysis, while for MID, negative correlation between state 2 and large-win trials was not replicated with FD as a regressor.

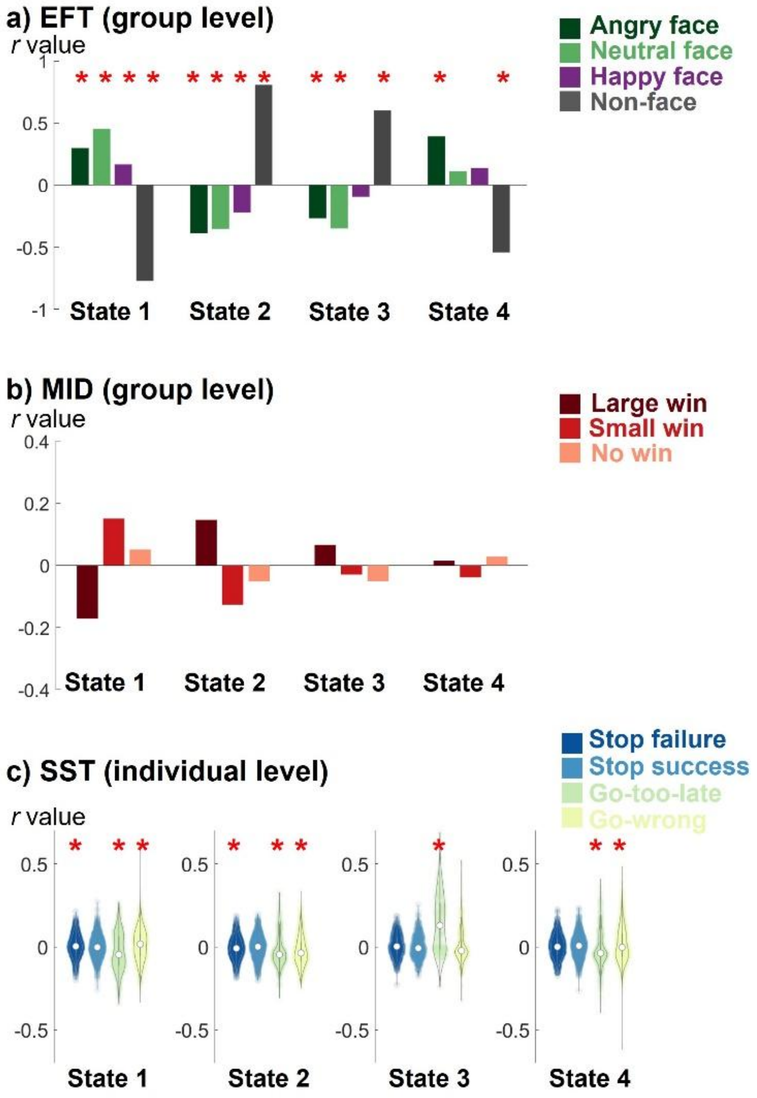

Table S1 A multi-step quality control pipeline for the resting-state and task-fMRI data

	REST	EFT	MID	SST
Data completion				
Missing neuroimaging	330	2	9	10
Missing demographics	0	18	18	18
Missing task onset file	0	12	48	23
Imaging quality control				
Visual QC	21	76	76	76
Incorrect scanning duration	0	2	5	2
Excessive motion (mean FD > 0.2 mm)	75	66	66	98
Normalization failure (group mask $r < 0.85$)	0	0	0	0
Included participants	991	1263	1221	1218

Table S2 Demographic information of included IMAGEN participants

	REST (*n* = 991)	EFT (*n* = 1263)	MID (*n* = 1221)	SST (*n* = 1218)
Age	19.01 ± 0.72	19.08 ± 0.76	19.08 ± 0.75	19.08 ± 0.76
Female (%)	523 (52.77)	666 (52.73)	646 (52.91)	639 (52.46)
Num. of subjects from each recruitment site	115 / 116 / 166 / 56 / 123 / 149 / 132 / 134	190 / 128 / 164 / 101 / 172 / 154 / 154 / 200	186 / 112 / 161 / 101 / 168 / 152 / 146 / 195	187 / 122 / 163 / 96 / 171 / 140 / 147 / 192

Table S3 Region label and peak coordinates of the 61 selected components

IC	Domain	Region	Abbreviation	MNI_X	MNI_Y	MNI_Z
39	SCN*	Thalamus	Thal	14.5	-21.5	16.5
46	SCN	Putamen	PUT	-21.5	7.5	0.5
73	SCN	Caudate (head)	CAU.h	15.5	25.5	12.5
84	SCN	Caudate (body)	CAU.b	-12.5	-4.5	15.5
92	SCN	Nucleus accumbens	NAc	5.5	17.5	0.5
67	AUD	Superior temporal gyrus	STG	59.5	-21.5	1.5
74	AUD	L Temporal pole	L.TP	-47.5	8.5	-31.5
80	AUD	L Posterior Superior temporal gyrus	L.STG.pos	-51.5	-46.5	4.5
96	AUD	R Anterior Middle temporal gyrus	R.MTG.ant	51.5	-0.5	-23.5
97	AUD	R Temporal pole	R.TP	44.5	7.5	-37.5
99	AUD	R Posterior Middle temporal gyrus	R.STG.pos	57.5	-48.5	13.5
3	SMN	Primary motor cortex	PMC	-2.5	-25.5	69.5
4	SMN	Premotor cortex	Premotor	-54.5	-9.5	31.5
10	SMN	L Precentral gyrus	L.preCG	-38.5	-27.5	64.5
15	SMN	R Precentral gyrus	R.preCG	39.5	-22.5	58.5
19	SMN	Paracentral gyrus	paraCG	-3.5	-25.5	60.5
24	SMN	Superior parietal lobe	SPL	-21.5	-49.5	67.5
32	SMN	Supplementary motor area	SMA	-21.5	-9.5	67.5
63	SMN	Cingulate motor area	CMA	3.5	10.5	42.5
68	SMN	Frontal eye field	FEF	45.5	-4.5	49.5
72	SMN	Postcentral gyrus	postCG	57.5	-21.5	40.5
76	SMN	Rolandic Operculum	ROper	50.5	-30.5	22.5
77	SMN	L Inferior Parietal Lobe	L.IPL	-44.5	-40.5	45.5
98	SMN	Intraparietal sulcus	IPS	29.5	-66.5	48.5
16	VSN	Inferior Occipital Gyrus	IOG	-26.5	-96.5	-4.5
30	VSN	Calcarine	CAL	2.5	-82.5	1.5
33	VSN	Lingual gyrus	LG	15.5	-61.5	6.5
38	VSN	Fusiform anterior	FG.ant	27.5	-42.5	-11.5
47	VSN	Superior Occipital Gyrus	SOG	-20.5	-90.5	22.5
49	VSN	Fusiform posterior	FG.pos	26.5	-70.5	-10.5
86	VSN	Middle Occipital Gyrus	MOG	-30.5	-75.5	9.5
89	VSN	Visual motion area	VMA	47.5	-67.5	-2.5
28	CON	Frontal Pole	FP	27.5	61.5	0.5

IC	Domain	Region	Abbreviation	MNI_X	MNI_Y	MNI_Z
34	CON	Superior Frontal Gyrus	SFG	-15.5	16.5	64.5
35	CON	Medial Superior Frontal Gyrus	SFG.med	-20.5	32.5	40.5
43	CON	Medial prefrontal cortex	mPFC	-0.5	58.5	25.5
54	CON	L Inferior Frontal gyrus (pars triangularis)	L.IFG.tri	-51.5	25.5	6.5
56	CON	Insula posterior	INS.pos	-41.5	5.5	-11.5
58	CON	Middle Frontal gyrus	MFG	33.5	47.5	33.5
61	CON	Inferior Frontal gyrus (pas Opercularis)	IFG.oper	-45.5	17.5	30.5
64	CON	Middle Cingulate cortex	MCC	2.5	-25.5	30.5
70	CON	R Inferior Frontal gyrus (pars triangularis)	R.IFG.tri	39.5	44.5	-1.5
71	CON	Insula anterior	INS.ant	35.5	25.5	3.5
83	CON	Hippocampus	Hipp	-23.5	-21.5	-14.5
85	CON	R Inferior Parietal lobe / Middle Frontal gyrus	R.IPL.MFG	51.5	-55.5	48.5
87	CON	R Inferior Frontal gyrus	R.IFG	57.5	11.5	10.5
90	CON	Parahippocampal	paraHipp	-23.5	-10.5	-26.5
94	CON	Amygdala	AMY	30.5	11.5	-25.5
95	CON	L Inferior Parietal lobe / Middle Frontal gyrus	L.IPL.MFG	-45.5	-64.5	45.5
31	DMN	Orbitofrontal cortex	OFC	-12.5	41.5	-16.5
37	DMN	Precuneus	PCu	-6.5	-60.5	61.5
42	DMN	Anterior Cingulate Cortex	ACC	-6.5	-60.5	61.5
75	DMN	Posterior Cingulate / Medial Orbital Frontal cortex	PCC.mPFC	-2.5	41.5	6.5
88	DMN	Precuneus / Posterior Cingulate	PCu.PCC	9.5	-69.5	36.5
2	CER	Cerebellum 1	CER1	12.5	-64.5	-44.5
6	CER	L Cerebellum Crus	L.CER.crus	-35.5	-60.5	-40.5
11	CER	Cerebellum 2	CER2	14.5	-57.5	-34.5
12	CER	R Cerebellum Crus	R.CER.crus	38.5	-58.5	-41.5
22	CER	Cerebellum Crus2	CER.crus2	23.5	-84.5	-34.5
25	CER	Vermis	Vermis	5.5	-58.5	-17.5
62	CER	Cerebellum 3	CER3	12.5	-70.5	-31.5

*Sixty-one ICA components were categorized into seven domains: subcortical (SCN), temporal (TEP), sensorimotor (SMN), visual (VSN), cognitive control (CON), default mode (DMN) and cerebellar (CEB) networks, based on their function and anatomical locations.

Table S4 One-way ANOVA and post-hoc t-test comparisons of network modularity and participation coefficients across the four FNC states

	Modularity	Participation (positive)	Participation (negative)
ANOVA	**F = 37.81** **p = 2.16e-6**	**F = 22.13** **p = 3.52e-5**	**F = 34.38** **p = 3.58e-6**
State 1 vs. 2	**t = -12.64,** **p = 0.001**	**t = 14.03,** **p = 7.84e-4**	**t = 21.11,** **p = 2.32e-4**
State 1 vs. 3	**t = -13.75,** **p = 8.33e-4**	**t = 45.64** **p = 2.32e-5**	**t = 33.47** **p = 5.86e-5**
State 1 vs. 4	**t = -8.35,** **p = 0.004**	**t = 4.39** **p = 0.02**	**t = 5.49,** **p = 0.01**
State 2 vs. 3	t = -3.42, p = 0.04	t = 2.84, p = 0.07	t = 2.44, p = 0.09
State 2 vs. 4	t = -0.04, p = 0.97	t = 0.31, p = 0.78	t = -0.19, p = 0.86
State 3 vs. 4	t = 1.51, p = 0.23	t = -0.70, p = 0.53	t = -1.07, p = 0.36

Two-sided p-values are reported. Statistically significant differences after FDR correction (q <0.05) are marked in **BOLD**.

Table S5 Spatial correlations (Pearson's *r*) between FNC states with different sliding window lengths from 8.8s (4TR) to 70.4s (32TR) with FNC states from 17.6s (8TR)

	8.8s (4TR)				35.2s (16TR)				70.4s (32TR)			
REST	FNC 1	FNC 2	FNC 3	FNC 4	FNC 1	FNC 2	FNC 3	FNC 4	FNC 1	FNC 2	FNC 3	FNC 4
State1	1.00*	0.76	0.70	0.78	1.00	0.81	0.75	0.85	0.99	0.85	0.79	0.89
State2	0.77	1.00	0.64	0.64	0.81	1.00	0.69	0.66	0.85	0.99	0.73	0.68
State3	0.71	0.65	1.00	0.53	0.72	0.66	1.00	0.62	0.71	0.67	0.99	0.66
State4	0.81	0.64	0.55	1.00	0.80	0.69	0.60	0.99	0.80	0.73	0.64	0.96
EFT	FNC 1	FNC 2	FNC 3	FNC 4	FNC 1	FNC 2	FNC 3	FNC 4	FNC 1	FNC 2	FNC 3	FNC 4
State1	1.00	0.79	0.62	0.81	0.99	0.84	0.65	0.86	0.98	0.88	0.70	0.90
State2	0.81	0.99	0.67	0.69	0.84	0.98	0.68	0.74	0.85	0.97	0.70	0.78
State3	0.62	0.65	1.00	0.61	0.63	0.69	1.00	0.63	0.62	0.69	0.99	0.65
State4	0.80	0.65	0.59	0.99	0.80	0.70	0.64	0.99	0.80	0.74	0.69	0.98
MID	FNC 1	FNC 2	FNC 3	FNC 4	FNC 1	FNC 2	FNC 3	FNC 4	FNC 1	FNC 2	FNC 3	FNC 4
State1	1.00	0.81	0.82	0.83	0.99	0.82	0.83	0.86	0.98	0.86	0.87	0.90
State2	0.80	1.00	0.73	0.65	0.82	0.99	0.76	0.72	0.84	0.98	0.79	0.76
State3	0.81	0.73	1.00	0.65	0.83	0.75	0.99	0.71	0.83	0.79	0.97	0.76
State4	0.82	0.65	0.64	1.00	0.80	0.65	0.65	0.98	0.81	0.67	0.68	0.97
SST	FNC 1	FNC 2	FNC 3	FNC 4	FNC 1	FNC 2	FNC 3	FNC 4	FNC 1	FNC 2	FNC 3	FNC 4
State1	1.00	0.80	0.63	0.80	1.00	0.84	0.66	0.85	0.99	0.88	0.71	0.88
State2	0.80	1.00	0.56	0.68	0.83	1.00	0.58	0.72	0.85	0.98	0.62	0.76
State3	0.63	0.55	1.00	0.54	0.63	0.58	1.00	0.58	0.63	0.60	0.99	0.60
State4	0.81	0.68	0.55	1.00	0.82	0.73	0.59	1.00	0.82	0.76	0.64	0.98

*Bold number indicates the highest spatial correlations between FNC of different window lengths and FNC states using window length of 17.6s.

Table S6 Spatial correlation (Pearson's *r* value) between FNC states with different *k*-means clustering numbers

	k = 2		*k* = 3			*k* = 5				
REST	FNC1	FNC2	FNC1	FNC2	FNC3	FNC1	FNC2	FNC3	FNC4	FNC5
State1	0.97*	0.74	0.98	0.80	0.72	0.99	0.78	0.65	0.79	0.83
State2	0.87	0.72	0.75	1.00	0.66	0.79	1.00	0.68	0.65	0.63
State3	0.69	0.99	0.69	0.65	1.00	0.65	0.64	0.98	0.55	0.95
State4	0.88	0.60	0.89	0.71	0.57	0.82	0.65	0.54	1.00	0.62
EFT	FNC1	FNC2	FNC1	FNC2	FNC3	FNC1	FNC2	FNC3	FNC4	FNC5
State1	0.98	0.79	0.99	0.77	0.81	0.99	0.79	0.62	0.79	0.89
State2	0.82	0.94	0.86	0.94	0.68	0.82	1.00	0.65	0.67	0.73
State3	0.63	0.86	0.62	0.87	0.63	0.65	0.67	1.00	0.60	0.51
State4	0.89	0.75	0.80	0.68	1.00	0.82	0.67	0.60	1.00	0.69
MID	FNC1	FNC2	FNC1	FNC2	FNC3	FNC1	FNC2	FNC3	FNC4	FNC5
State1	0.98	0.83	0.99	0.80	0.83	0.99	0.76	0.82	0.80	0.88
State2	0.75	0.98	0.78	0.98	0.66	0.79	0.97	0.65	0.63	0.86
State3	0.81	0.84	0.87	0.83	0.65	0.82	0.80	0.99	0.62	0.61
State4	0.91	0.67	0.79	0.63	1.00	0.83	0.61	0.64	1.00	0.69
SST	FNC1	FNC2	FNC1	FNC2	FNC3	FNC1	FNC2	FNC3	FNC4	FNC5
State1	0.98	0.83	0.98	0.83	0.65	0.99	0.79	0.62	0.79	0.87
State2	0.79	0.98	0.78	1.00	0.58	0.81	0.99	0.55	0.67	0.78
State3	0.64	0.70	0.63	0.58	1.00	0.65	0.59	1.00	0.56	0.47
State4	0.91	0.74	0.91	0.74	0.58	0.82	0.67	0.55	1.00	0.74

*Bold number indicates the highest spatial correlation between FNC from *k* = 2~5 with FNC states from *k* = 4.

Table S7 Correlation coefficients between FNC state occurrences with ongoing task events

	State 1	State 2	State 3	State 4
EFT (group-level)*				
Angry faces	***r* = 0.27, *p* = 2.00e-4**	***r* = -0.43, *p* < 1.00e-4**	*r* = -0.13, *p* = 0.09	***r* = 0.41, *p* < 1.00e-4**
Neutral faces	***r* = 0.39, *p* < 1.00e-4**	***r* = -0.44, *p* < 1.00e-4**	*r* = -0.13, *p* = 0.08	*r* = 0.16, *p* = 0.03
Happy faces	*r* = 0.15, *p* = 0.05	***r* = -0.26, *p* < 1.00e-4**	*r* = -0.004, *p* = 0.96	*r* = 0.16, *p* = 0.03
Non-face	***r* = -0.61, *p* = 7.88e-20**	***r* = 0.85, *p* = 1.51e-52**	***r* = 0.20, *p* = 0.007**	***r* = -0.55, *p* = 7.70e-16**
MID (group-level)				
Large win	*r* = 0.17, *p* = 0.05	***r* = -0.20, *p* = 0.008**	*r* = 0.11, *p* = 0.15	*r* = -0.001, *p* = 0.99
Small win	*r* = -0.12, *p* = 0.12	*r* = 0.14, *p* = 0.07	*r* = -0.01, *p* = 0.84	*r* = -0.04, *p* = 0.56
No win	*r* = -0.06, *p* = 0.43	*r* = 0.06, *p* = 0.43	*r* = -0.06, *p* = 0.42	*r* = 0.03, *p* = 0.68
SST (individual-level)				
Stop success	***t* = 2.60, *p* = 0.01**	***t* = -4.60, *p* = 4.63e-6**	*t* = 1.92, *p* = 0.06	*t* = 0.32, *p* = 0.75
Stop failure	*t* = -0.47, *p* = 0.64	*t* = -1.12, *p* = 0.26	*t* = -0.46, *p* = 0.64	*t* = 2.10, *p* = 0.04
Go too-late	***t* = -7.04, *p* = 5.32e-12**	***t* = -8.50, *p* = 1.51e-16**	***t* = 15.86, *p* = 2.88e-43**	***t* = -4.01, *p* = 6.72e-5**
Go wrong	***t* = 4.32, *p* = 1.72e-5**	***t* = -10.33, *p* = 5.45e-24**	*t* = -0.59, *p* = 0.56	***t* = 4.75, *p* = 2.27e-6**

*For EFT and MID tasks, experimental designs are the same across all participants, thus we calculate Pearson's correlation between occurrence of task events and group-averaged FNC state occurrence (two-sided test). For SST, stimuli onsets differ across subjects. We therefore calculated partial correlations between state occurrence and task stimuli at the individual subject level. Correlation coefficients were compared to 0 using a one-sample *t*-test to determine their significance (two-sided test).

FDR significant (q <0.05) correlations are shown in **BOLD**.

Table S8 Comparison of correlation coefficients between FNC state occurrences and task events

	State 1	State 2	State 3	State 4
EFT (group-level)*				
Angry vs Non-faces	z = 9.13, p = 0.0000	z = -18.61, p = 0.0000	z = -2.71, p = 0.007	z = 10.31, p = 0.0000
Neutral vs Non-faces	z = 11.12, p = 0.0000	z = -18.95, p = 0.0000	z = -2.71, p = 0.007	z = 6.79, p = 0.0000
Happy vs Non-faces	z = 7.62, p = 0.0000	z = -14.22, p = 0.0000	z = -1.64, p = 0.10	z = 6.86, p = 0.0000
Angry vs Neutral faces	z = -1.20, p = 0.23	z = 0.10, p = 0.92	z = 0.00, p = 1.00	z = 2.39, p = 0.02
Happy vs Neutral faces	z = -2.28, p = 0.02	z = 1.78, p = 0.08	z = 1.15, p = 0.25	z = 0.00, p = 1.00
Angry vs Happy faces	z = 1.10, p = 0.27	z = -1.67, p = 0.09	z = -1.15, p = 0.25	z = 2.40, p = 0.02
MID (group-level)				
Large-win vs small-win	z = 2.35, p = 0.02	z = -2.79, p = 0.005	z = 0.96, p = 0.34	z = 0.32, p = 0.75
Large-win vs no-win	z = 1.85, p = 0.06	z = -2.10, p = 0.04	z = 1.35, p = 0.18	z = -0.24, p = 0.81
Small-win vs no-win	z = -0.48, p = 0.63	z = 0.64, p = 0.52	z = 0.40, p = 0.69	z = -0.55, p = 0.58
SST (individual-level)				
Stop-success vs Stop-failure	t = 2.18, p = 0.03	t = -2.34, p = 0.02	t = 1.82, p = 0.07	t = -1.29, p = 0.20
Stop-success vs Go too-late	t = 6.92, p = 0.0000	t = 5.12, p = 0.0000	t = -14.46, p = 0.0000	t = 3.70, p = 0.0002
Stop-success vs Go wrong	t = -2.29, p = 0.02	t = 6.17, p = 0.0000	t = 1.60, p = 0.11	t = -3.85, p = 0.0001
Stop-failure vs Go too-late	t = 5.97, p = 0.0000	t = 5.99, p = 0.0000	t = -15.78, p = 0.0000	t = 4.98, p = 0.0000
Stop-failure vs Go wrong	t = -3.82, p = 0.0001	t = 7.69, p = 0.0000	t = 0.18, p = 0.85	t = -3.05, p = 0.002
Go too-late vs Go wrong	t = -6.51, p = 0.0000	t = -1.18, p = 0.24	t = 14.82, p = 0.0000	t = -6.35, p = 0.0000

*For EFT and MID tasks, correlation coefficients were compared using Pearson and Filon's z-statistic (two-sided test). For SST, correlation coefficients between state occurrence and task stimuli were calculated at an individual subject level and compared between conditions using two-sided one-sample t-test.

FDR significant (q <0.05) correlations are shown in **BOLD**.

Table S9 The 29 behavioural items on reinforcement-related psychopathology and cognition.

No.	Abbreviation	Questionnaire	Item	Included in STRATIFY[10]
1	DAWBASpePho	DAWBA [1]	Specific Phobia	No
2	DAWBASocPho	DAWBA	Social Phobia	No
3	DAWBAAgoPho	DAWBA	Agoraphobia	No
4	DAWBAOCD	DAWBA	Obsessive compulsive disorder	No
5	DAWBAGenAnx	DAWBA	Generalized anxiety disorder	No
6	DAWBADep	DAWBA	Depression	No
7	DAWBAADHD	DAWBA	Attention deficit hyperactivity	No
8	DAWBAED	DAWBA	Eating disorder	No
9	SDQEmotion	SDQ [2]	Emotional symptoms	Yes
10	SDQConduct	SDQ	Conduct problems	Yes
11	SDQHyper	SDQ	Hyperactivity/inattention	Yes
12	SDQPeer	SDQ	Peer relationship problems	Yes
13	ADRSDep	ADRS [3]	Depression	No
14	ESPADSmokFreq	ESPAD [4]	Smoke frequency last 30 days	Yes
15	ESPADDrinkOccasions	ESPAD	Drink occasions last 12 months	Yes
16	ESPADDrinkFiveMore	ESPAD	Binge drinking last 12 months	Yes
17	ESPADDrunk	ESPAD	Drunk last 12 months	Yes
18	AUDITTotal	AUDIT [5]	Alcohol Use	Yes
19	SURPSHopelessness	SURPS [6]	Negative Thinking	Yes
20	SURPSAnxietySensitivity	SURPS	Anxiety Sensitivity	Yes
21	SURPSImpulsivity	SURPS	Impulsivity	Yes
22	SURPSSensationSeeking	SURPS	Sensation Seeking	Yes
23	KirbyTotal	MCQ [7]	Reward delay discounting	Yes
24	AGNLatencyNeg	AGN [8]	Response latency to negative words	No
25	CGTDelayAversion	CGT [9]	Delay aversion	Yes
26	CGTDeliberationTime	CGT	Decision time	Yes
27	CGTQualityOfDecisionMaking	CGT	Quality of decision-making	Yes
28	CGTRiskAdjustment	CGT	Risk Adjustment	Yes
29	CGTRiskTaking	CGT	Risk Taking	Yes

[1] Development and Well-Being Assessment (DAWBA) [4]

[2] Strengths and Difficulties Questionnaire (SDQ) (66)
[3] Adolescent Depression Rating Scale (ADRS) [6]
[4] European School Survey Project on Alcohol and Other Drugs (ESPAD) [7]
[5] Alcohol Use Disorders Identification Test (AUDIT) [8]
[6] Substance Use Risk Profile Scale (SURPS) [9]
[7] Monetary-Choice Questionnaire (MCQ) [10]
[8] Affective Go-Nogo task (AGN) (CANTAB, www.cambridgecognition.com)
[9] Cambridge Gambling Task (CGT) (CANTAB, www.cambridgecognition.com)
[10] Among the 29 behavioural items used in IMAGEN, 19 were available in STRATIFY cohort

Table S10 Sparse partial least squares (sPLS) analysis* on the FNC state dwell time and reinforcement-related behaviours

	Training set correlation	Test set correlation	Test set p-value
REST			
State 1	0.17	0.10	0.14
State 2	0.10	0.08	0.18
State 3	0.19	0.25	0.002
State 4	0.14	0.12	0.08
EFT			
State 1	0.13	0.20	0.004
State 2	0.14	0.10	0.10
State 3	0.16	0.17	0.01
State 4	0.17	0.14	0.03
MID			
State 1	0.24	0.09	0.11
State 2	0.20	0.15	0.02
State 3	0.06	0.10	0.10
State 4	0.23	0.16	0.01
SST			
State 1	0.11	0.18	0.01
State 2	0.17	0.22	0.001
State 3	0.18	0.28	0.0003
State 4	0.18	0.13	0.04

*A multiple hold-out sPLS analysis was performed using the CCA/PLS Toolkit [5]. Data were split into a training and test set (20%), and the training set was further divided into a training and validation set (20%) to select the best hyperparameter (L1 penalty). Statistical significance of the test set correlation was determined by 5,000 times permutation inference (two-sided, uncorrected $p < 0.05$) (Material and Methods).

Table S11 Task specific FNC and their *t*-statistics to distinguish condition of interests in each task*

FNC Label	t_EFT	t_MID	t_SST
EFT specific connectivity			
PCu.PCC - L.STG.pos	14.02	-1.77	-0.53
ACC - L.STG.pos	11.98	0.72	1.04
VMA - FG.ant	11.94	-1.22	2.00
MCC - L.STG.pos	10.69	0.11	-0.84
PCu.PCC - STG	10.48	-1.85	-1.54
PCu.PCC - R.STG.pos	9.73	-1.63	-2.97
MFG - L.STG.pos	9.13	-0.94	0.35
PCu - L.STG.pos	8.97	0.17	0.53
ACC - R.STG.pos	8.85	0.65	-4.92
PCu - IFG.oper	8.50	-1.40	2.63
LG - R.STG.pos	8.41	1.78	-4.55
LG - L.STG.pos	8.22	1.88	-2.11
PCC.mPFC - R.MTG.ant	8.07	-1.67	-0.36
MFG - L.IFG.tri	7.90	3.24	-0.42
MFG - STG	7.86	-0.01	-1.64
ACC - R.MTG.ant	7.62	-3.91	-0.88
MFG - R.STG.pos	7.51	2.27	4.45
IFG.oper - FG.ant	7.43	-1.03	-0.10
MCC - STG	7.41	-2.04	-0.42
PCu.PCC - IFG.oper	7.35	0.36	0.24
MID specific connectivity			
PCC.mPFC - R.IPL.MFG	-4.81	8.62	-3.20
ACC - IPS	-10.53	8.10	-0.58
MCC - IFG.oper	4.41	7.92	-0.36
ACC - R.IPL.MFG	-2.44	7.67	-3.05
PCC.mPFC - L.IPL.MFG	-3.64	7.44	-4.48
ACC - IFG.oper	3.39	6.80	-2.69
PCC.mPFC - IFG.oper	6.58	6.57	-2.16
PCC.mPFC - FP	-6.87	6.22	-6.22
R.IPL.MFG - MCC	-1.84	5.97	-0.78
mPFC - postCG	0.85	5.91	0.57
L.preCG - R.STG.pos	-3.45	5.82	-0.31

PCu.PCC - postCG	-3.77	5.73	1.02
PCC.mPFC - R.IFG.tri	-2.77	5.62	-3.21
OFC - L.IPL.MFG	-0.52	5.60	-1.81
ACC - FP	-3.63	5.58	-5.27
L.IPL.MFG - FG.ant	-1.57	5.50	-2.74
L.IPL.MFG - LG	-2.18	5.39	-1.88
OFC - R.IPL.MFG	-1.93	5.35	-0.55
SFG.med - IPS	-4.01	5.31	0.11
ACC - R.IFG.tri	-1.83	5.26	-3.66
SST specific connectivity			
PCC.mPFC - R.Oper	3.37	-1.10	6.80
PCC.mPFC - postCG	1.24	1.99	6.41
PCC.mPFC - L.preCG	0.35	0.28	6.31
ACC - L.preCG	-2.35	0.50	6.03
ACC - R.Oper	1.16	-1.25	5.87
PCC.mPFC - R.preCG	0.23	1.38	5.51
ACC - R.preCG	-1.86	-0.09	5.45
R.preCG - STG	0.07	0.48	5.23
PCC.mPFC - Premotor	1.76	2.05	5.08
Premotor - R.MTG.ant	0.63	0.45	4.77
R.Oper - L.TP	-0.06	0.42	4.67
R.IPL.MFG - PUT	0.67	-2.27	4.61
L.IPL.MFG - R.IFG	1.39	-0.63	4.55
MFG - R.STG.pos	7.51	2.27	4.45
mPFC - R.preCG	2.21	0.87	4.44
ACC - postCG	-3.03	1.78	4.44
Premotor - L.TP	0.59	0.26	4.44
mPFC - R.Oper	1.37	0.56	4.40
ACC - Premotor	0.21	1.87	4.27
PCC.mPFC - L.IPL	0.71	1.46	4.20

* Correlation coefficients between each FNC strength and task conditions were calculated, and then compared between conditions using one-sample *t*-tests (20 FNC with the highest *t*-statistics were shown for each task). Correlation coefficients were compared between angry faces versus neutral faces for the EFT, large-win versus no-win condition for the MID and stop-success versus stop-failure condition for the SST to identify FNC that are related to emotional processing, reward sensitivity and motor inhibition.

Table S12 Regression of 29 behavioural items on time-varying FNC and static FNC in IMAGEN. Model significance is obtained by comparing the full model with null models (only constant term) using likelihood ratio test (uncorrected $p_{\text{one-tail}}$<0.05).

Behaviour	FNC	R2(%)	R2adj(%)	Log-likelihood	p_model
EFT_SDQConduct	full model	1.00	0.59	-1979.71	0.03
	time-varying FNC	0.98	0.65	-1979.85	0.02
	static FNC	0.09	0.01	-1985.30	0.30
EFT_SDQPeer	full model	0.80	0.39	-2108.15	0.08
	time-varying FNC	0.71	0.39	-2108.68	0.07
	static FNC	0.07	-0.01	-2112.62	0.36
EFT_ESPADSmokFreq	full model	0.99	0.59	-2339.80	0.03
	time-varying FNC	0.97	0.64	-2339.94	0.02
	static FNC	0.35	0.27	-2343.77	0.04
EFT_ESPADDrinkOccasions	full model	0.99	0.58	-2392.00	0.03
	time-varying FNC	0.71	0.38	-2393.75	0.07
	static FNC	0.21	0.13	-2396.78	0.10
EFT_ESPADDrinkFiveMore	full model	2.15	1.75	-2477.48	0.00
	time-varying FNC	1.33	1.00	-2482.65	0.00
	static FNC	0.72	0.64	-2486.37	0.00
EFT_ESPADDrunk	full model	0.88	0.48	-2424.91	0.05
	time-varying FNC	0.73	0.40	-2425.85	0.06
	static FNC	0.12	0.04	-2429.58	0.22
EFT_AUDITTotal	full model	1.18	0.75	-1540.11	0.02
	time-varying FNC	1.12	0.78	-1540.43	0.01
	static FNC	0.02	-0.06	-1546.78	0.61
EFT_SURPSAnxietySensitivity	full model	1.12	0.71	-781.01	0.02
	time-varying FNC	0.56	0.23	-784.50	0.15
	static FNC	0.22	0.14	-786.54	0.10
EFT_SURPSImpulsivity	full model	1.39	0.98	-670.01	0.00
	time-varying FNC	1.01	0.68	-672.32	0.01
	static FNC	0.31	0.23	-676.59	0.05
EFT_SURPSSensationSeeking	full model	1.00	0.59	-905.42	0.03
	time-varying FNC	0.55	0.22	-908.14	0.15

	static FNC	0.36	0.28	-909.32	0.04
	full model	0.92	0.49	2575.30	0.06
EFT_KirbyTotal	time-varying FNC	0.91	0.57	2575.27	0.03
	static FNC	0.39	0.31	2572.21	0.03
	full model	1.00	0.59	-7249.86	0.03
EFT_AGNLatencyNeg	time-varying FNC	0.85	0.52	-7250.78	0.03
	static FNC	0.60	0.52	-7252.33	0.01
	full model	1.14	0.74	708.38	0.01
EFT_CGTDelayAversion	time-varying FNC	1.04	0.72	707.79	0.01
	static FNC	0.28	0.20	703.00	0.06
	full model	0.83	0.41	-92.96	0.08
MID_DAWBAOCD	time-varying FNC	0.80	0.46	-93.13	0.05
	static FNC	0.01	-0.07	-97.80	0.71
	full model	0.98	0.55	-2587.24	0.04
MID_SDQEmotion	time-varying FNC	0.86	0.53	-2587.91	0.04
	static FNC	0.64	0.55	-2589.24	0.01
	full model	0.81	0.38	-651.73	0.09
MID_SURPSImpulsivity	time-varying FNC	0.79	0.45	-651.88	0.05
	static FNC	0.00	-0.08	-656.48	0.82
	full model	0.84	0.40	2515.24	0.09
MID_KirbyTotal	time-varying FNC	0.54	0.18	2513.52	0.20
	static FNC	0.69	0.60	2514.38	0.01
	full model	0.85	0.42	-536.96	0.07
SST_DAWBASocPho	time-varying FNC	0.59	0.25	-538.50	0.14
	static FNC	0.05	-0.04	-541.69	0.46
	full model	0.90	0.48	-752.93	0.06
SST_DAWBAED	time-varying FNC	0.87	0.53	-753.13	0.04
	static FNC	0.20	0.12	-757.04	0.12
	full model	1.05	0.63	-2299.74	0.03
SST_ESPADDrinkOccasions	time-varying FNC	0.61	0.27	-2302.35	0.12
	static FNC	0.10	0.01	-2305.39	0.28
SST_ESPADDrinkFiveMore	full model	0.85	0.43	-2395.74	0.07
	time-varying FNC	0.53	0.20	-2397.65	0.18

	static FNC	0.15	0.06	-2399.93	0.18
SST_SURPSImpulsivity	full model	1.04	0.62	-641.48	0.03
	time-varying FNC	0.84	0.50	-642.69	0.04
	static FNC	0.01	-0.07	-647.56	0.71
SST_CGTRiskTaking	full model	0.95	0.53	852.74	0.04
	time-varying FNC	0.80	0.46	851.84	0.05

Table S13 Demographic information of STRATIFY participants

	Alcohol use disorder (AUD, *n* = 125)	Major depressive disorder (MDD, *n* = 131)	Healthy controls (CON, *n* = 183)	*p**	*F* or *chi2*
Age	22.19±2.06	22.25±2.26	22.11±1.27	0.80	0.23
Sex (M/F)	49/76	31/100	68/115	**0.01**	**8.58**
Recruitment sites	5/82/38	10/71/50	41/93/49	**5.42e-06**	**29.78**

* Group-comparisons are performed using one-way ANOVA test (Age) and chi-square tests (Sex, Recruitment sites). Two-sided *p*-values are reported. Statistically significant differences after FDR correction ($q < 0.05$) are marked in BOLD.

Table S14 Replication of correlation between FNC states dwell time with SST go too-late error in the STRATIFY cohort. Spearman's rank correlation (two-sided) was performed as task performance do not follow normal distribution.

	**State 1**	**State 3**
Healthy controls (CON)	***rho* = -0.26, *p* = 0.01**	***rho* = 0.31, *p* = 0.003**
Major depressive disorder (MDD)	*rho* = -0.14, *p* = 0.25	***rho* = 0.28, *p* = 0.02**
Alcohol use disorder (AUD)	*rho* = -0.05, *p* = 0.70	***rho* = 0.34, *p* = 0.004**

FDR significant (*q* <0.05) correlations are shown in **BOLD**. Note that the *p*-values are larger than correlations in the IMAGEN cohort probably due to differences in sample size (IMAGEN *n*=1218; STRATIFY CON, MDD and AUD *n*=183, 131, 125). Yet Spearman's *rho* was in the same direction as the IMAGEN cohort.

Supplementary References

1. Grosbras, M. H. & Paus, T. Brain networks involved in viewing angry hands or faces. *Cereb. Cortex* **16**, 1087–1096 (2006).
2. Knutson, B., Fong, G. W., Adams, C. M., Varner, J. L. & Hommer, D. Dissociation of reward anticipation and outcome with event-related fMRI. *Neuroreport* **12**, 3683–3687 (2001).
3. Bari, A. & Robbins, T. W. Inhibition and impulsivity: Behavioral and neural basis of response control. *Prog. Neurobiol.* **108**, 44–79 (2013).
4. Goodman, R., Ford, T., Richards, H., Gatward, R. & Meltzer, H. The Development and Well-Being Assessment: Description and initial validation of an integrated assessement of child and adolescent psychopathology. *J. Child Psychol. Psychiatry Allied Discip.* (2000) doi:10.1017/S0021963099005909.
5. Goodman, R. Psychometric properties of the strengths and difficulties questionnaire. *J. Am. Acad. Child Adolesc. Psychiatry* **40**, 1337–45 (2001).
6. Revah-Levy, A., Birmaher, B., Gasquet, I. & Falissard, B. The Adolescent Depression Rating Scale (ADRS): a validation study. *BMC Psychiatry* **7**, 1–10 (2007).
7. Hibell, B. *et al. The ESPAD Report 2003*. *Drugs* (2004).
8. Saunders, J. B., Aasland, O. G., Babor, T. F., de la Fuente, J. R. & Grant, M. Development of the Alcohol Use Disorders Identification Test (AUDIT): WHO Collaborative Project on Early Detection of Persons with Harmful Alcohol Consumption--II. *Addiction* **88**, 791–804 (1993).
9. Woicik, P. A., Stewart, S. H., Pihl, R. O. & Conrod, P. J. The substance use risk profile scale: A scale measuring traits linked to reinforcement-specific substance use profiles. *Addict. Behav.* **34**, 1042–1055 (2009).
10. Kirby, K. N., Petry, N. M. & Bickel, W. K. Heroin addicts have higher discount rates for delayed rewards than non-drug-using controls. *J. Exp. Psychol. Gen.* **128**, 78–87 (1999).
