# Supplementary material for: Neurocognitive characterization of behaviour and mental illness through time-varying brain network analysis
Source: Nat Commun. 2026 Feb 3;17:1353. doi: 10.1038/s41467-025-67398-w (PMC12877127; doi:10.1038/s41467-025-67398-w)
Supplement: Supplementary file 2 — Reporting Summary [file 41467_2025_67398_MOESM2_ESM.pdf]

nature portfolio

Corresponding author(s): Gunter Schumann
Xiao Chang

Last updated by author(s): 24/3/25

Reporting Summary

Nature Portfolio wishes to improve the reproducibility of the work that we publish. This form provides structure for consistency and transparency in reporting. For further information on Nature Portfolio policies, see our Editorial Policies and the Editorial Policy Checklist.

Statistics

For all statistical analyses, confirm that the following items are present in the figure legend, table legend, main text, or Methods section.

n/a	Confirmed	
☐	☒	The exact sample size (*n*) for each experimental group/condition, given as a discrete number and unit of measurement
☐	☒	A statement on whether measurements were taken from distinct samples or whether the same sample was measured repeatedly
☐	☒	The statistical test(s) used AND whether they are one- or two-sided<br>*Only common tests should be described solely by name; describe more complex techniques in the Methods section.*
☐	☒	A description of all covariates tested
☐	☒	A description of any assumptions or corrections, such as tests of normality and adjustment for multiple comparisons
☐	☒	A full description of the statistical parameters including central tendency (e.g. means) or other basic estimates (e.g. regression coefficient) AND variation (e.g. standard deviation) or associated estimates of uncertainty (e.g. confidence intervals)
☐	☒	For null hypothesis testing, the test statistic (e.g. *F*, *t*, *r*) with confidence intervals, effect sizes, degrees of freedom and *P* value noted<br>*Give P values as exact values whenever suitable.*
☒	☐	For Bayesian analysis, information on the choice of priors and Markov chain Monte Carlo settings
☒	☐	For hierarchical and complex designs, identification of the appropriate level for tests and full reporting of outcomes
☐	☒	Estimates of effect sizes (e.g. Cohen's *d*, Pearson's *r*), indicating how they were calculated

Our web collection on statistics for biologists contains articles on many of the points above.

Software and code

Policy information about availability of computer code

Data collection	This study analyzed neuroimaging and behavioural data from two large-scale, multi-center, neuroimaging-genetic datasets: a population-based IMAGEN cohort (Schumann, G. et al. 2010. Mol. Psychiatry 15, 1128–1139) and a clinical STRATIFY cohort (Xie, C. et al. 2023. Nat. Med. 29, 1232–1242) including patients with depression and alcohol use disorder.
Data analysis	T1-weighted MRI data were preprocessed using Freesurfer v6.0<br>Resting-state fMRI data were preprocessed using FSL version 5.0.9 and ANTs version 1.9.2. Task-based fMRI data were processed using SPM12.<br>Group independent component analysis and time-varying functional network connectivity (FNC) estimation were performed using GIFT software (https://trendscenter.org/software/gift/).<br>Statistical analyses were performed using Matlab 2020b.<br>The core code used to run the analyses reported in this study can be found at: https://github.com/xchang007/tvFNC_IMAGEN.git

For manuscripts utilizing custom algorithms or software that are central to the research but not yet described in published literature, software must be made available to editors and reviewers. We strongly encourage code deposition in a community repository (e.g. GitHub). See the Nature Portfolio guidelines for submitting code & software for further information.

Data

Policy information about availability of data

All manuscripts must include a data availability statement. This statement should provide the following information, where applicable:

- Accession codes, unique identifiers, or web links for publicly available datasets
- A description of any restrictions on data availability
- For clinical datasets or third party data, please ensure that the statement adheres to our policy

IMAGEN and STRATIFY data are available on request from https://imagen-project.org/.

Data and materials availability statement:
Requests for materials should be addressed to Gunter Schumann. The core code used to run the analyses reported in this study can be found at: https://github.com/xchang007/tvFNC_IMAGEN.git

Research involving human participants, their data, or biological material

Policy information about studies with human participants or human data. See also policy information about sex, gender (identity/presentation), and sexual orientation and race, ethnicity and racism.

Reporting on sex and gender	Sex was taken as a covariate throughout the study. The population-based IMAGEN cohort has a balanced male (48.27%) and female (51.73%) participants. The clinical STRATIFY cohort has 125 (74.40%) female participants in major depressive disorder group, 88 (58.67%) female participants in alcohol use disorder group, and 123 (58.57%) female participants in healthy control group.
Reporting on race, ethnicity, or other socially relevant groupings	Participants of the IMAGEN and STRATIFY cohort were recruited from United Kingdom, German, France and Ireland. The datasets recruited Caucasian participants.
Population characteristics	The primary sample is the IMAGEN dataset. We analyzed neuroimaging and behavioural data from 1417 participants at mean age of 19.09 ± 0.76 years old, including 733 female subjects (51.73%). Participants were recruited from eight research centers in the United Kingdom, German, France and Ireland.<br>We validated our findings in the STRATIFY cohort, including alcohol use disorder (AUD, n = 150), major depressive disorder (MDD, n = 168) and healthy controls (CON, n = 210) from STRATIFY. Demographic information was provided in Table S12.
Recruitment	The IMAGEN cohort recruited 2,000 14-year-old adolescents from eight research centers in the United Kingdom, German, France and Ireland. Participants were followed up at 16, 19 and 23 years-old.<br>STRATIFY cohort recruited patients (ages 19-25) with alcohol use disorder, major depression and healthy controls from three recruitment sites in Berlin, London and Southampton, which are also recruitment sites for IMAGEN.
Ethics oversight	Ethics approval was obtained at each recruitment site by local research ethics committee. Written consent was obtained from each participant.

Note that full information on the approval of the study protocol must also be provided in the manuscript.

Field-specific reporting

Please select the one below that is the best fit for your research. If you are not sure, read the appropriate sections before making your selection.

- [x] Life sciences
- [ ] Behavioural & social sciences
- [ ] Ecological, evolutionary & environmental sciences

For a reference copy of the document with all sections, see nature.com/documents/nr-reporting-summary-flat.pdf

Life sciences study design

All studies must disclose on these points even when the disclosure is negative.

Sample size	The primary sample IMAGEN dataset recruited 1417 participants at 19 years old. This sample size can detect any effect size (cohen's d) > 0.16, with two-tailed significance level of 0.001, power 0.85.
Data exclusions	We performed a multi-step quality control for neuroimaging data (Table S1): excluding participants with missing information, failed visual QC, incorrect scanning duration, excessive head motion (mean framewise displacement (FD) > 0.2 mm), and unsuccessful normalization (correlation with group mask r < 0.85). In total, we analyzed data from 991 subjects with resting-state fMRI, 1263 participants with emotional faces task (EFT), 1221 with monetary-incentive delay task (MID) and 1218 subjects with stop-signal task (SST) (Table S2).
Replication	We replicated the FNC states using the resting-state and task-fMRI data fron the STRATIFY sample (Figure S14). For the correlation with behaviours, we first replicated the higher association with time-varying FNC than static FNC in STRATIFY healthy controls (Figure 6c) and then generalized the finding by distinguishing between patients and controls (Figure 6d).
Randomization	We did not use randomization in the study, as this study does not control for any particular variable.

Blinding	Blinding was not applicable to this study as this study is observational.

Reporting for specific materials, systems and methods

We require information from authors about some types of materials, experimental systems and methods used in many studies. Here, indicate whether each material, system or method listed is relevant to your study. If you are not sure if a list item applies to your research, read the appropriate section before selecting a response.

Materials & experimental systems

n/a	Involved in the study
☒	☐ Antibodies
☒	☐ Eukaryotic cell lines
☒	☐ Palaeontology and archaeology
☒	☐ Animals and other organisms
☒	☐ Clinical data
☒	☐ Dual use research of concern
☒	☐ Plants

Methods

n/a	Involved in the study
☒	☐ ChIP-seq
☒	☐ Flow cytometry
☐	☒ MRI-based neuroimaging

Plants

Seed stocks	*Report on the source of all seed stocks or other plant material used. If applicable, state the seed stock centre and catalogue number. If plant specimens were collected from the field, describe the collection location, date and sampling procedures.*
Novel plant genotypes	*Describe the methods by which all novel plant genotypes were produced. This includes those generated by transgenic approaches, gene editing, chemical/radiation-based mutagenesis and hybridization. For transgenic lines, describe the transformation method, the number of independent lines analyzed and the generation upon which experiments were performed. For gene-edited lines, describe the editor used, the endogenous sequence targeted for editing, the targeting guide RNA sequence (if applicable) and how the editor was applied.*
Authentication	*Describe any authentication procedures for each seed stock used or novel genotype generated. Describe any experiments used to assess the effect of a mutation and, where applicable, how potential secondary effects (e.g. second site T-DNA insertions, mosiacism, off-target gene editing) were examined.*

Magnetic resonance imaging

Experimental design

Design type	Three task-based fMRI: emotional faces task (EFT), monetary-incentive delay task (MID), stop-signal task (SST) and resting-state fMRI
Design specifications	The EFT was adapted from Grosbras and Paus (2006) to assess social-emotional processing (Fig. S1). Participants were instructed to watch a 18s block of either face (angry, happy, neutral) or non-face (control) stimuli. Each block of face stimuli comprises black and white video clips (2-5 s) of one type of face in movement (three males and three females). The control stimuli block consists of black and white concentric circles expanding or contracting at various speeds, roughly matching the contrast and motion of the face clips. Each type of face stimuli was repeated four times and intermixed with twelve blocks of the control stimuli.<br><br>The MID was modified based on Knutson et al., (2001) to investigate neural mechanism of reward processing. Scanning session consists of 42 10s trials for every participant. Each trial begins with an anticipatory cue (250 ms), indicating participant can win 10 points (large-win), or 2 points (small-win) or no points (no-win) if responded correctly. After a variable delay (4,000-4,500 ms) of fixation on a white cross-hair, a target (250-400 ms) appear on the left or right side of the screen the same as the cue. Participants were instructed to press the left or right button using the index finger corresponding to the side of target as soon as possible.<br><br>The SST was designed to measure neural response of inhibition control. Participants were instructed to press button using the left or right index finger corresponding to go signals (an arrow pointing to left or right, 60 trials), and withhold their responses to unpredictable stop signals (an arrow pointing upwards immediately followed the go signals, 300 trials).
Behavioral performance measures	Task performances include: MID reaction time (RT) and accuracy (ACC) for large-win, small-win and no-win trials; SST go signal RT, stop signal RT, stop failure error, go too-late error, go wrong error.<br><br>Bbehaviour questionnaires assess mental disorder symptoms, substance use and neuropsychological functions, including 29 items (Table S8) from:<br>1 Development and Well-Being Assessment (DAWBA)<br>2 Strengths and Difficulties Questionnaire (SDQ)

3 Adolescent Depression Rating Scale (ADRS)
4 European School Survey Project on Alcohol and Other Drugs (ESPAD)
5 Alcohol Use Disorders Identification Test (AUDIT)
6 Substance Use Risk Profile Scale (SURPS)
7 Monetary-Choice Questionnaire (MCQ)
8 Affective Go-Nogo task (AGN) (CANTAB, www.cambridgecognition.com)
9 Cambridge Gambling Task (CGT) (CANTAB, www.cambridgecognition.com)

Acquisition

Imaging type(s): T1-weighted structural MRI; task-based and resting-state fMRI

Field strength: 3T

Sequence & imaging parameters: For each participant, we collected a high-resolution T1-weighted MRI (T1) scan using the Magnetization Prepared Rapid Acquisition Gradient Echo (MPRAGE) sequence. Blood-oxygen-level-dependent (BOLD) functional images were acquired with gradient-echo, echo-planar imaging (EPI) sequence during resting-state and three cognitive tasks: emotional faces task (EFT), monetary-incentive delay task (MID) and stop-signal task (SST). Scanning parameters of T1-weigthed images are: repetition time (TR) = 2,300 ms; echo time (TE) = 2.8 ms; flip angle (FA) = 8°; isotropic voxel size 1.1 mm; 256×256×160 matrix; sagittal slice plane. Acquisition parameters of functional images are: TR = 2,200 ms, TE = 30 ms, FA = 75°; 64×64×40 matrix, voxel size=3.4×3.4×2.4 mm, slice gap = 1mm.

Area of acquisition: Whole brain

Diffusion MRI: ☐ Used ☒ Not used

Preprocessing

Preprocessing software: Resting-state fMRI data were pre-processed with FMRIBs Software Library (FSL version 5.0.9) and Advanced Normalization Tools (ANTs version 1.9.2). Non-brain tissue was removed (FSL BET) and images were corrected for head motion (FSL MCFLIRT), then spatially smoothed using a 4mm FWHM Gaussian kernel. In addition, artifact components were removed for each data set using an automatic classification algorithm (ICA-AROMA v0.3). The resulting cleaned data set was detrended and normalized to MNI standard space using the custom EPI template (ANTs). Pre-processed data were resliced to 3mm isotropic voxels.
Task-based fMRI scans were pre-processed using Statistical Parametric Mapping (SPM12, http://www.fil.ion.ucl.ac.uk/spm/). Pre-processing steps include: non-brain tissue removal, slice-timing correction, head movement realignment using a rigid body transformation, and images were non-linearly warped on the MNI space using a customized EPI template. Normalized images were smoothed using 5mm FWHM Gaussian kernel. Pre-processed data were resliced to 3mm isotropic voxels.

Normalization: Images were non-linearly warped on the MNI space using a customized EPI template.

Normalization template: Customized EPI template created based on an average of mean images of 400 adolescents.

Noise and artifact removal: see above

Volume censoring: see above

Statistical modeling & inference

Model type and settings: FNC states were extracted using k-means clustering analysis.
Correlation between FNC state dwell time and behaviours were performed using sparse Partial Least Square (sPLS) analysis (https://github.com/anaston/cca_pls_toolkit) and hold-out validation.
Group comparisons were performed using ANOVA and two-sample t-tests
Association were performed using Pearson's correlation and Spearman's rank correlation when variables do not follow normal distribution.
Behavioural variances explained was quantified using linear regression model.

Effect(s) tested: F-test and t-test statistics; correlation coefficients (r values); variance explained (R-squared)

Specify type of analysis: ☒ Whole brain ☐ ROI-based ☐ Both

Statistic type for inference (See Eklund et al. 2016): Functional connectivity was calculated between brain regions derived from group independent component analysis.

Correction: Multiple testing was corrected using the false discovery rate (FDR) at p<0.05

Models & analysis

n/a	Involved in the study
☒	☐ Functional and/or effective connectivity
☒	☐ Graph analysis
☒	☐ Multivariate modeling or predictive analysis
